# TMEM131‐Mediated Soluble TRAIL Triggered Type II Alveolar Epithelial Cell Senescence in Radiation‐Induced Lung Injury

**DOI:** 10.1002/advs.202509973

**Published:** 2025-11-26

**Authors:** Linzhi Han, Chunsheng Wang, Xiuli Guo, Yuan Luo, Jianguo Zhang, Fajian He, Zihang Zeng, Jiang Luo, Wen Ouyang, Yan Gong, Conghua Xie

**Affiliations:** ^1^ Department of Pulmonary Oncology Zhongnan Hospital of Wuhan University Wuhan 430071 China; ^2^ Department of Radiology Zhongnan Hospital of Wuhan University Wuhan 430071 China; ^3^ Tumor Precision Diagnosis and Treatment Technology and Translational Medicine Hubei Engineering Research Center Zhongnan Hospital of Wuhan University Wuhan 430071 China; ^4^ Hubei Key Laboratory of Tumor Biological Behaviors Zhongnan Hospital of Wuhan University Wuhan 430071 China; ^5^ Wuhan Research Center for Infectious Diseases and Cancer Chinese Academy of Medical Sciences Wuhan 430071 China

**Keywords:** cellular senescence, DR5/mTOR, RILI, soluble TRAIL, TMEM131

## Abstract

Radiation‐induced lung injury (RILI) limits radiotherapy dose for thoracic tumors. It is currently urgent to clarify RILI pathogenesis and find safe and effective therapeutical strategy. Transcriptomics of RILI mouse lungs indicate that cellular senescence is substantially involved in RILI pathogenesis, and anti‐senescence compounds alleviate RILI and pulmonary inflammatory. Single‐cell RNA sequencing and multi‐color immunofluorescence demonstrate that type II alveolar epithelial cells (AECIIs) are the main senescent cells, and quantitative proteomics illustrate that tumor necrosis factor‐related apoptosis‐inducing ligand (TRAIL) is more secreted. Clinical samples also confirms that the plasma levels of TRAIL are significantly increased in RILI patients. Mechanistically, AECII‐specific TRAIL knockout (*Trail^f/f^;Sftpc‐Cre*) mice presents reduced cellular senescence and RILI, accompanied by reduced mitophagy. Soluble TRAIL activates the mTOR pathway through death receptor 5 to hinder mitophagy, resulting in impaired mitochondrial accumulation and cellular senescence. Immunoprecipitation mass spectrometry identifies that endoplasmic reticulum (ER)‐localized transmembrane molecule TMEM131 interact with TRAIL and mediates its transportation from ER to Golgi through Sec23 homolog A of the coat protein complex II. Interruption of this transportation process led to ER‐associated degradation of TRAIL proteins through ubiquitylation. The results indicate the pro‐senescent role of TRAIL during RILI pathogenesis and reveals the TMEM131‐mediated intricate secretory process of TRAIL.

## Graphical abstract

TMEM131 recruits the COPII complex to accelerate TRAIL transportation from endoplasmic reticulum to Golgi apparatus, and promotes soluble TRAIL secretion. TRAIL inhibits mitophagy and induces senescence through DR5 receptor in type II alveolar epithelial cells, ultimately driving radiation‐induced lung injury (RILI) progression. This study reveals TRAIL's key role in mitophagy and cellular senescence, offering a potential therapeutic target for RILI.

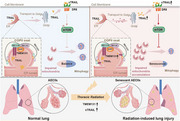

## 1 Introduction

Radiation‐induced lung injury (RILI) limits the curative effects of radiotherapy for thoracic tumors and induces pulmonary tissue injury. Approximately 50% patients receiving thoracic radiotherapy would develop RILI, severely influencing prognosis and life equality of patients.^[^ ^1^, ^2^, ^3^ ^]^ Early RILI manifests as pneumonitis, while late RILI is characterized by lung fibrosis and irreversible pulmonary dysfunction.^[^ ^4^ ^]^ The pathogenesis of RILI is complicated and remains unclear. Amifostine and hormone analogues are cytoprotective agents against ionizing radiation, accompanied by adverse effects such as vomiting, hypotension and osteoporosis.^[^ ^5^, ^6^, ^7^, ^8^ ^]^ Currently, there are no clinically approved drugs that are both effective and safe to alleviate RILI,^[^ ^9^ ^]^ making early intervention and development of novel targets for RILI extremely imperative.

The pulmonary epithelium consists of alveolar type 1 epithelial cells (AT1) with air change ability and type 2 alveolar epithelial cells (AECIIs) responsible for differentiation and regeneration.^[^ ^10^ ^]^ AECIIs, marked by surfactant protein C (SFTPC), are the main responders to lung injury and damage repairing, reconstructing alveolar structure via differentiating or transforming into other cell types (AT1 or fibroblasts).^[^ ^11^, ^12^, ^13^ ^]^ AECIIs also display extraordinary sensitivity to cell senescence in various lung disorders including idiopathic pulmonary fibrosis.^[^ ^11^, ^14^, ^15^, ^16^, ^17^, ^18^, ^19^, ^20^ ^]^ Senescent AECIIs fail to differentiate into AT1, but transform into fibroblasts, accelerating fibrosis development.^[^ ^10^, ^21^, ^22^, ^23^ ^]^ Radiation‐induced cell senescence is often characterized by accumulated DNA damage,^[^ ^13^, ^24^ ^]^ subsequently activating the cellular DNA damage response ataxia telangiectasia mutated (ATM)/P53/P21 and/or Rb/P16 pathways, ultimately leading to permanent cell cycle arrest and cell senescence.^[^ ^25^ ^]^

Multiple studies indicated that cellular senescence was accompanied by mitophagy suppression, resulting in damaged mitochondria accumulation, suggesting that mitophagy is also an important indicator of cell senescence. In idiopathic pulmonary fibrosis, there is a substantial accumulation of dysfunctional mitochondria in AECIIs, which increases with age. Overexpression of mitophagy‐related molecules decreases mitochondrial damage and alleviates lung fibrosis.^[^ ^26^ ^]^ Mitophagy probably regulates RILI‐related AECII senescence as well. However, the detailed functions and underlying mechanisms are yet to be fully elucidated.

Senescent cells secrete various senescence‐associated secretory phenotype (SASP) proteins to extracellular matrix, inducing adjacent cell senescence. Moreover, SASP molecules recruit immune cells, facilitate fibroblast to myofibroblast transformation, and polarize macrophages into the fibrotic M2 phenotype,^[^ ^27^, ^28^ ^]^ thereby causing chronic inflammation.^[^ ^27^ ^]^ Although AECII senescence plays important roles in multiple types of lung fibrosis, there are few studies on its functions in RILI. Therefore, exploring the underlying mechanisms of AECII senescence and inflammatory responses in RILI might provide novel insights to alleviate adverse effects of thoracic radiotherapy.

Tumor necrosis factor (TNF)‐related apoptosis‐inducing ligand (TRAIL) is a type II trimeric transmembrane protein, which selectively induces tumor cell apoptosis and promotes inflammatory responses via activating pathways such as nuclear factor (NF)‐κB and c‐Jun N‐terminal kinase (JNK). TRAIL expression is significantly upregulated in the plasma of patients with chronic obstructive pulmonary disease,^[^ ^29^ ^]^ and TRAIL deficiency reduces lung inflammation and improved lung function.^[^ ^30^ ^]^ Human TRAIL receptors, including death receptor (DR) 4, DR5, decoy receptor (DCR) 1, DCR2 and osteoprotegerin (OPG),^[^ ^31^ ^]^ exhibit various functions in the development of asthma,^[^ ^32^ ^]^ lung fibrosis, allergic lung inflammation, and pulmonary hypertension.^[^ ^33^ ^]^ Mouse TRAIL receptors involve mDR5 and 3 decoy receptors (mDcR1, mDcR2 and mOPG). Simultaneously, blocking TRAIL can improve spatial learning and memory capabilities in mouse models with Alzheimer's disease, alleviate brain inflammation, and restore the function of brain parenchyma.^[^ ^34^ ^]^ These findings provide further evidence that TRAIL and its receptors might serve as key inflammatory factors in the respiratory diseases and aging.

In tumors and inflammatory diseases, TRAIL functions as a secretory protein with anti‐apoptotic and pro‐inflammatory effects. Despite numerous clinical studies on TRAIL, the effectiveness of related drugs is limited owing to the short half‐life and poor stability of TRAIL protein. Consequently, exploring the synthetic and secretory pathways of TRAIL may offer novel therapeutic strategies for both oncogenic and non‐oncogenic diseases. Newly synthesized polypeptides (cargo proteins) are initially processed through folding, assembly, and glycosylation in endoplasmic reticulum (ER). Correctly folded cargo proteins accumulate at specific regions of the endoplasmic reticulum, named ER exit sites, which are dynamic clusters of vesicles and tubules. ER exit sites are responsible for packaging proteins into coat protein II (COPII) vesicles, completing COPII assembly.^[^ ^35^, ^36^ ^]^ COPII vesicles, enclosing the cargo proteins, bud from ER membrane and transport to the Golgi apparatus, where they fuse into Golgi membrane. After further folding and maturation in Golgi, proteins are secreted to extracellular space through exocytosis. As an important vehicle for transporting unmature proteins, COPII is a bilayer vesicle. The inner layer consists of SEC23 and SEC24, which recognize and interact with the cargo proteins. while the outer layer is composed of SEC13 and SEC31 that form the membrane skeleton.^[^ ^37^, ^38^ ^]^ SEC23 is crucial in cargo selection, including collagen and lipids,^[^ ^39^, ^40^, ^41^ ^]^ and the expression levels of SEC23 are directly proportional to the intracellular protein transport rate.^[^ ^42^ ^]^ These studies indicated that SEC23 might be indispensable for cellular protein synthesis and delivery of secretory proteins.

Notably, the interaction between cargo proteins and COPII vesicle requires cargo receptors to regulate transport of cargo proteins. Researches revealed that thyroid adenoma‐associated gene assists in binding programmed death‐ligand 1(PD‐L1) to SEC24A‐associated COPII and increases PD‐L1 expression on cell membrane.^[^ ^43^ ^]^ Unc‐51‐like kinase 1 interacts with SEC23A and blocks the coupling of secretory protein cargos to COPII.^[^ ^44^ ^]^ cAMP response element‐binding protein regulated transcription coactivator 2 disrupts the SEC23A‐SEC31A interaction, thus decreasing COPII‐dependent sterol regulatory element‐binding protein 1 maturation.^[^ ^41^ ^]^ Collectively, cargo receptors are indispensable for delivering cargo proteins to COPII vesicles.

If COPII complex fails to carry cargo proteins or incorrectly processed proteins reside in ER, ER quality control mechanisms (ERQC) would be aroused, including ER‐associated protein degradation (ERAD), unfolded protein response (UPR), and autophagy. In ERAD pathway, ER degradation‐enhancing α‐mannosidase‐like protein (EDEM) would trim unqualified proteins, exposing α‐1,6 mannose residues, which target the E3 ubiquitin ligase HMG‐CoA reductase degradation protein 1 (HRD1) complex.^[^ ^45^ ^]^ Next, the HRD1 complex polyubiquitinates the proteins needing degradation.^[^ ^46^, ^47^ ^]^ Finally, substrates labeled with ubiquitin by E3 ubiquitin ligase move from ER to cytoplasm for proteasomal degradation. To date, no studies elucidated the transport process of TRAIL from the ER to Golgi. Understanding intracellular maturation process and degradation mechanism of TRAIL protein would control TRAIL secretion, providing alternative therapeutic target molecules for diseases.

Our research identifies the senescence of AECIIs as a critical pathogenic factor in RILI. Under the stimulation of radiation, TRAIL proteins secreted by AECIIs bind to DR5 receptor in an autocrine manner on the surface of AECIIs, further inactivating mTOR‐dependent mitophagy. Mitochondrial dysfunction leads to AECII senescence, ultimately accelerating RILI progression. Notably, we further investigated the key selective cargo protein receptor TMEM131 regulating TRAIL protein secretion. These results showed that upregulation of TMEM131 accelerated the transport of TRAIL protein in ER. Targeted intervention of TMEM131 in AECIIs effectively inhibits their senescence and RILI development. Rather than mass use of TRAIL antibodies, targeting TMEM131‐TRAIL secretion in AECIIs would be more accurate to treat RILI without affecting pro‐apoptosis TRAIL of immune cells. Our studies presented promising strategies to develop novel treatment strategies to target AECII senescence in RILI.

## 2 Results

### 2.1 TRAIL‐Mediated AECII Senescence Contributed to RILI Development

**Figure 1:**
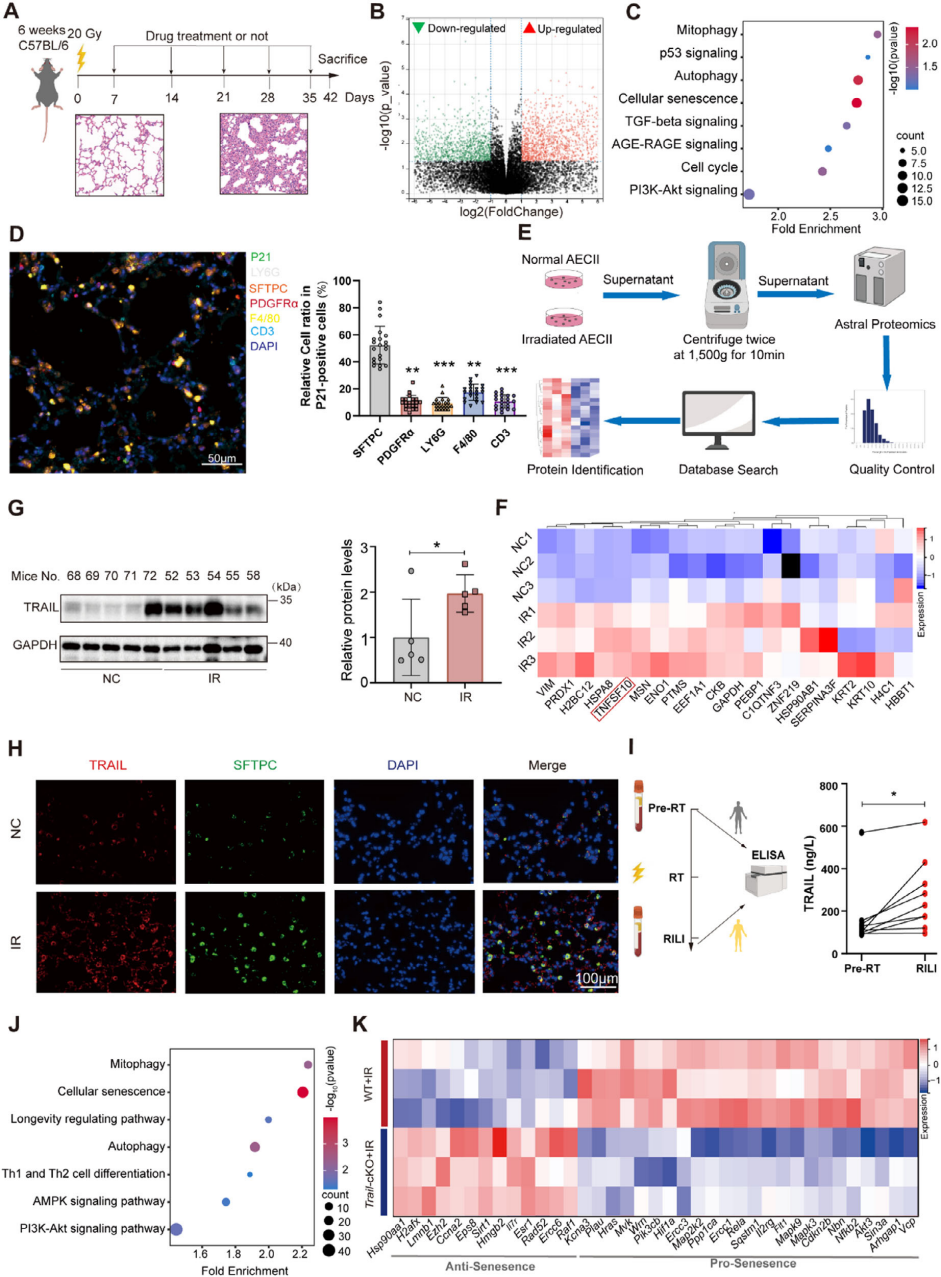
TRAIL deficiency in AECIIs alleviated its senescence and RILI progression. A) Schematic diagram of the RILI mouse model. B) Differentially‐expressed genes in the lung tissues between irradiated and control mice. C) Metascape enrichment analysis for transcriptomic sequencing of the lung tissues irradiated and control mice. D) Optimized multi‐color immunofluorescence of F4/80 (macrophage marker), platelet‐derived growth factor receptor α (PDGFRα, fibroblast marker), Sftpc (AECII marker), LY6G (neutrophil marker), CD3 (lymphocyte marker), P21 (cell senescence marker), DAPI (cell nucleus) in the lung tissues from irradiated mouse. E) Schematic diagram of the Astral quantitative proteomic analysis using the supernatants of normal and irradiated AECIIs. F) Differential proteins in the supernatants between normal and irradiated AECIIs. G) Immunoblotting of TRAIL in irradiated and control mouse lung tissues. H) Immunofluorescence of TRAIL and SFTPC in irradiated and control mouse lung tissues. I) ELISA for sTRAIL protein levels in human plasma samples. J) Metascape enrichment analysis for transcriptomic sequencing of the RILI tissues from WT and *Trail*‐cKO mice. K) Differentially‐expressed senescent genes in the RILI tissues between WT and *Trail*‐cKO mice. Data shown as mean ± SEM, N = 5–20; *p < 0.05; **p < 0.01; ***p < 0.001.

To examine the essential pathways during RILI development, we established the RILI mouse model via irradiating C57BL/6 mice (6 weeks) at 20 Gy and collected the lung tissues 6 weeks later. Hematoxylin‐eosin staining images for lung tissues showed pulmonary consolidation and aberrant lung structures after 6 weeks (**Figure** **1**A). Transcriptomic analysis and metascape enrichment analysis showed that cell senescence pathways were substantially induced in the RILI mice (Figure 1B,C). To confirm that cell senescence contributed to RILI, we treated the mice with anti‐senescence drugs dasatinib and quercetin (DQ), and collected the bronchoalveolar lavage fluid (BALF) for flow cytometry 6 weeks later (Figure S1A,B, Supporting Information). The number of inflammatory macrophages, lymphocytes and neutrophils were substantially increased in the RILI mice, while DQ significantly reversed this phenomenon (Figure S1C, Supporting Information). Moreover, immunohistochemistry of lung tissues also demonstrated that DQ alleviated lung consolidation, collagen deposition and macrophage infiltration (Figure S1D, Supporting Information). These results indicated that cell senescence promotes RILI development.

To identify the types of pulmonary cells involved in the RILI‐related cell senescence, we analyzed the single‐cell transcriptomic data of irradiated mouse lung tissues, and found that *Sftpc*+ AECIIs had a high senescent rate (Figure S1E, Supporting Information). Optimized multi‐color immunofluorescence also confirmed that the co‐localization of cell senescence marker P21 with AECII marker SFTPC was more than that of other cell types (Figure 1D; Figure S1F, Supporting Information), suggesting that AECII senescence was a main source of RILI progression.

To explore the secretory proteins involved in these cell behaviors, the astral quantitative proteomic analysis of the supernatants from normal and irradiated AECIIs was performed (Figure 1E). TRAIL protein levels were substantially increased in the supernatants from irradiated AECIIs (Figure 1F; Figure S2, Supporting Information). Immunoblotting of lung tissues from normal and RILI mice presented higher TRAIL protein levels in the irradiated lungs (Figure 1G). Immunofluorescent results also demonstrated that radiation induced the expression of TRAIL proteins in SFTPC‐positive AECIIs in the mouse lungs (Figure 1H). Moreover, we recruited RILI patients and collected their peripheral blood samples before radiotherapy and after diagnosis of RILI. ELISA results suggested their plasma levels of TRAIL were significantly increased after RILI development (Figure 1I).

To confirm the role of TRAIL in AECIIs during RILI development, we specifically knocked TRAIL out in AECIIs via crossing *Trail^f/f^* female with the *Sftpc‐Cre* male mice. Six weeks after 20 Gy thoracic irradiation, lung tissues from *Trail^f/f^;Sftpc‐Cre* (*Trail*‐cKO) and *Trail^f/f^* (WT) mice were obtained for RNA sequencing. Remarkably, specific TRAIL deficiency in AECIIs were closely associated with cell senescence and mitophagy pathways in the lungs (Figure 1J). The mRNA levels of senescence‐associated genes^[^ ^48^ ^]^ between the WT and *Trail*‐cKO RILI mice were illustrated in the heat map (Figure 1K). While anti‐senescence genes including sirtuin 1 (*Sirt1*) were induced, the pro‐senescence genes including cyclin‐dependent kinase 4 inhibitor B (*Cdkn2b*) were downregulated in the *Trail*‐cKO lungs. Taken together, these results indicated that TRAIL‐mediated AECII senescence contributed to RILI development.

### 2.2 AECII‐Specific TRAIL Deficiency Enhanced the Suppressive Effects of Anti‐Senescence Drugs on RILI

**Figure 2:**
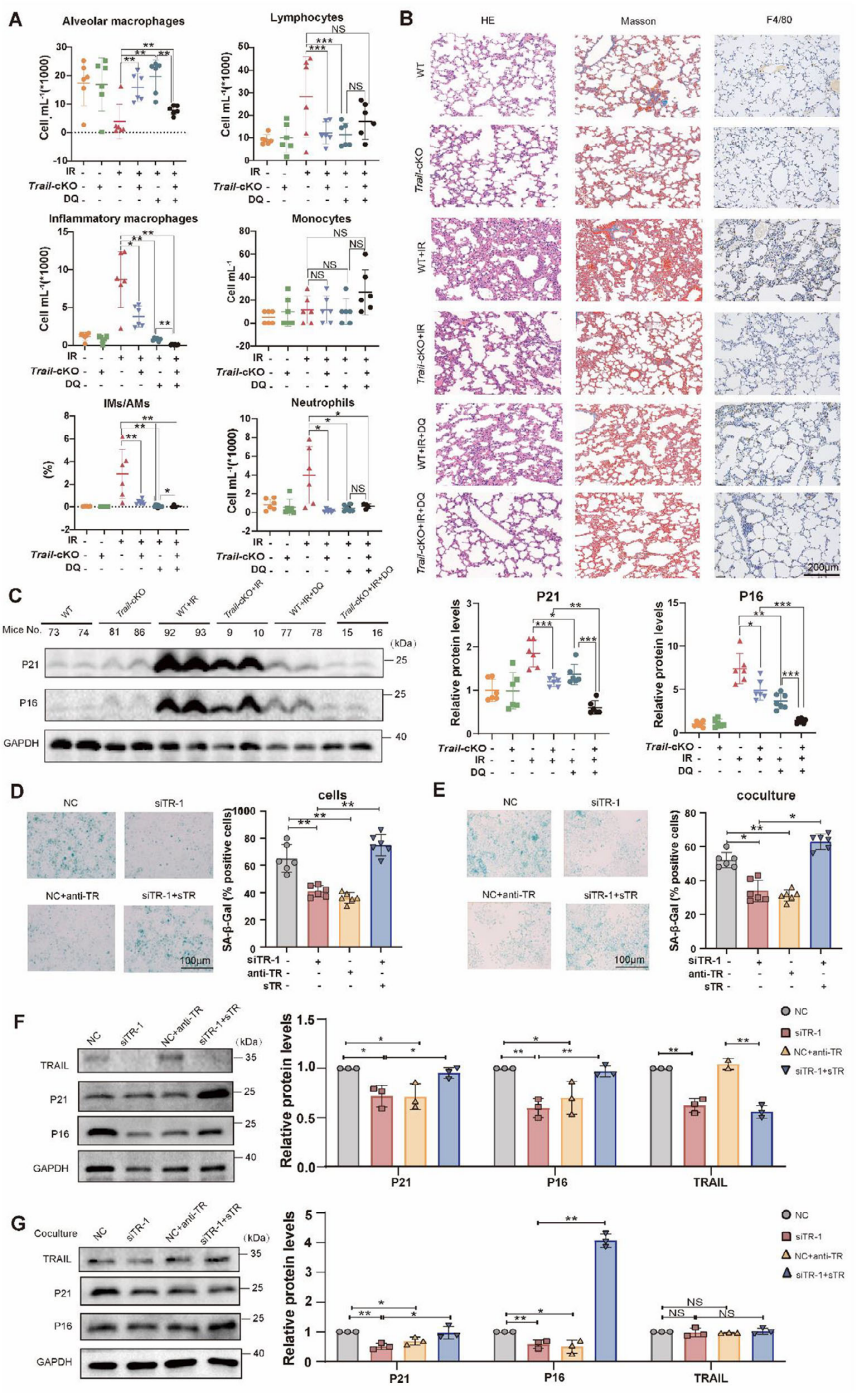
TRAIL downregulation reduced irradiation‐induced AECII senescence. A) Flow cytometry of infiltrated immune cells in BALF from WT and *Trail*‐cKO mice with and without irradiation and cell senescence inhibition. B) Representative histological staining of normal and irradiated lung tissues from WT and *Trail*‐cKO mice with and without cell senescence inhibition. C) Protein expression of cell senescence molecules. D) β‐galactosidase staining with anti‐TR or sTR in irradiated AECIIs. E) β‐galactosidase staining with anti‐TR or sTR in primary AECIIs cocultured with supernatant from irradiated AECIIs. F) Protein expression of cell senescence‐related molecules with TRAIL antibody or TRAIL recombinant protein in primary AECIIs. G) Protein expression of cell senescence‐related molecules with anti‐TR or sTR in primary AECIIs cocultured with supernatant from irradiated AECIIs. IMs/AMs, Inflammatory Macrophages/Alveolar Macrophages; anti‐TR, TRAIL antibody; sTR, TRAIL recombinant protein; siTR‐1, siRNA‐1 for TRAIL. Data shown as mean ± SEM, N = 3–6; *p < 0.05; **p < 0.01; ***p < 0.001; NS, *p* ≥ 0.05.

To confirm that TRAIL from AECIIs correlate with cell senescence and RILI progression, AECII‐specific TRAIL knockout mice were treated with anti‐senescence drugs DQ. Flow cytometry of BALF demonstrated that TRAIL deficiency in AECIIs improved the downregulation of inflammatory macrophages by anti‐senescence drugs (**Figure** **2**A; Figure S3A, Supporting Information). Immunohistochemistry also indicated that AECII‐specific TRAIL loss promoted the alleviative effects of DQ on lung consolidation, collagen deposition and macrophage infiltration in the RILI mice (Figure 2B). Immunoblotting illustrated that TRAIL knockout in AECIIs diminished radiation‐induced cell senescence marker P21 and P16 protein levels in the RILI lung tissues, and increased the inhibitory effects of anti‐senescence drugs on these proteins (Figure 2C). Moreover, AECII‐specific TRAIL deficiency strengthened the descendent impact of DQ on the mRNA levels of senescent and inflammatory cytokines interleukin (*Il*) 1b, *Il6*, *Il10*, *Il13*, transforming growth factor (*Tgf*) b1 and *Tnfa* (Figure S3B, Supporting Information). These results suggested that absence of TRAIL in AECIIs suppressed radiation‐induced lung inflammation and enhanced the therapeutic effects of anti‐senescence drugs.

To clarify the role of TRAIL in cell senescence, we downregulated TRAIL in both mouse primary AECIIs and alveolar epithelium MLE‐12 cells by small interference RNAs. TRAIL knockdown significantly reduced senescent rates in irradiated AECIIs and MLE‐12 cells (Figure S4A, Supporting Information). Immunoblotting confirmed the decreased protein levels of TRAIL and senescent markers in both cells (Figure S4B, Supporting Information). In addition, TRAIL silencing significantly downregulated senescent and inflammatory cytokines in irradiated MLE‐12 cells (Figure S4C, Supporting Information). Since TRAIL was identified from the supernatant proteins by proteomic analysis, we further examined the effects of secreted TRAIL on cell senescence. While TRAIL antibodies had familiar effects with TRAIL knockdown, recombinant TRAIL proteins reversed the inhibitory effects of TRAIL downregulation on radiation‐induced AECII senescence (Figure 2D). Both TRAIL knockdown in the irradiated AECIIs and addition of TRAIL blocking antibodies in their conditioned medium induced less senescence in the unirradiated AECIIs, while addition of soluble TRAIL in the conditioned medium from TRAIL knockdown AECIIs revoked senescence in the naive AECIIs, suggesting TRAIL increased irradiation‐induced AECII senescence in a paracrine manner (Figure 2E). AECIIs expressed less senescent markers in the TRAIL silencing and antibody groups, but recombinant TRAIL proteins rescued the decreased P21 and P16 protein levels induced by TRAIL deficiency (Figure 2F). P21 and P16 protein levels were downregulated in native AECIIs exposed to medium from irradiated AECIIs following TRAIL silencing and antibody treatment (Figure 2G). Moreover, the supernatant from TRAIL‐deficient AECIIs also induced less senescent and fibrogenic proteins in primary lung fibroblasts (Figure S4D, Supporting Information). Collectively, our findings suggested that TRAIL deficiency in AECIIs suppressed RILI via downregulating cell senescence.

### 2.3 TRAIL Promoted IR‐Induced Cell Senescence via Inhibiting Mitophagy through DR5

**Figure 3:**
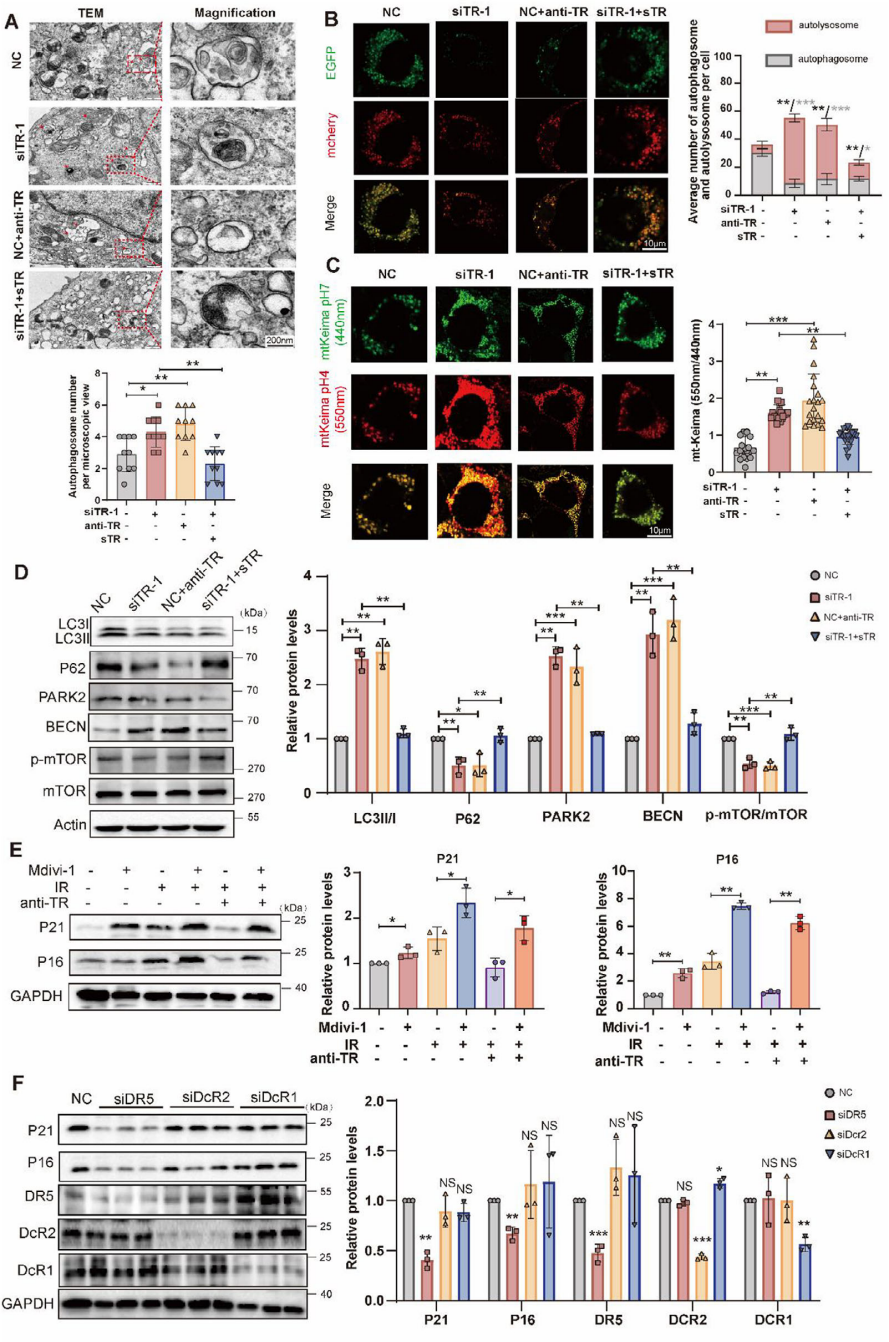
TRAIL promoted irradiation‐induced senescence via inhibiting mitophagy through DR5 in AECIIs. A) Representative electron microscopy images for mitophagosome. B) MLE‐12 cells were infected with mCherry‐EGFP‐LC3B adenovirus. C) MLE‐12 cells were infected with mtKeima fluorescent protein. D) Protein expression of mitophagy‐related molecules. E) Immunoblots of cell senescence‐related proteins with mitophagy inhibitors in MLE‐12 cells. F) Immunoblotting of cell senescence‐related molecules and TRAIL receptors after knocking down receptors. anti‐TR, TRAIL antibody; sTR, TRAIL recombinant protein; siTR‐1, siRNA‐1 for TRAIL; siDR5, siRNAs for mDR5; siDcR2, siRNAs for mDcTRAILR2; siDcR1, siRNAs for mDcTRAILR1. Data shown as mean ± SEM, N = 3–20; *p < 0.05; **p < 0.01; ***p < 0.001; NS, *p* ≥ 0.05.

Our transcriptome data revealed that AECII‐specific TRAIL deficiency in RILI lungs influenced mitophagy (Figure 1J). Mitochondria function as the major energy providers and essential regulatory machines for cellular homeostasis. Their dysfunction triggers a cascade of changes such as oxidative stress and cell senescence.^[^ ^49^ ^]^ Mitochondrial membrane potential change is an obvious indicant of mitophagy. The results of JC‐1 mitochondrial membrane potential flow cytometry indicated that TRAIL silencing and antibodies significantly altered JC‐1 distribution, whereas recombinant TRAIL proteins reversed these effects (Figure S5A, Supporting Information). Transmission electron microscopy illustrated more mitochondrial autophagosomes in the TRAIL deficiency and blockade groups, relapsed by exogenous TRAIL supply (**Figure** **3**A). To further verify whether TRAIL regulated mitophagy flux, mCherry‐EGFP‐LC3B adenovirus and mtKeima probe experiment was performed. Fluorescent images demonstrated that TRAIL downregulation exhibited accumulation of red puncta (Figure 3B,C), indicating improved mitophagy flux. Considering mitophagy process consist of mitophagosome formation and mitolysosome fusion, the expression levels of mitophagy flux‐related proteins were next assessed. Mitophagy‐initiation protein Beclin1, formation protein LC3BII/I and mitophagy marker PARK2 were upregulated, while mitophagy degradation marker P62 was downregulated in the TRAIL deficient group (Figure 3D). mTOR participates in the entire mitophagy process via inhibiting the initiation, intermediate and terminated steps, and might also mediate TRAIL‐suppressed mitophagy. Immunoblotting showed that TRAIL induced the protein levels of phosphorylation mTOR versus mTOR (Figure 3D), suggesting that TRAIL knockdown both increased mitophagosome formation and mitolysosome fusion via blocking mTOR signaling pathway. Bafilomycin A1 (BAF), an inhibitor for autophagosomes‐lysosomes fusion, enhanced LC3BII/I and P62 expression in all groups. These results also revealed that blocking TRAIL increased mitophagy flux on the basis of irradiation (Figure S5B, Supporting Information). Moreover, mitochondrial division dynamin‐related protein 1 (DRP1) inhibitor (Mdivi‐1), as a mitophagy inhibitor, upregulated both P21 and P16 (Figure 3E), indicating the indispensable role of mitophagy in TRAIL‐mediated cell senescence. Since TRAIL receptors have multiple functions, we further identified the specific receptor for TRAIL‐induced cell senescence. Only DR5 deficiency rescued induced P21 and P16 protein levels (Figure 3F). Lysosome‐associated genes were also upregulated in TRAIL deficiency or DR5 depletion cohorts, which were responsible for autophagosome‐lysosome fusion (Figure S5C, Supporting Information). Therefore, DR5 was involved in the TRAIL‐regulated cell senescence. Overall, these findings suggested that silencing TRAIL could mitigate irradiation‐induced cell senescence via stimulating mitophagy.

### 2.4 TMEM131 Interacted with TRAIL and was Associated with TRAIL‐Mediated AECII Aging

**Figure 4:**
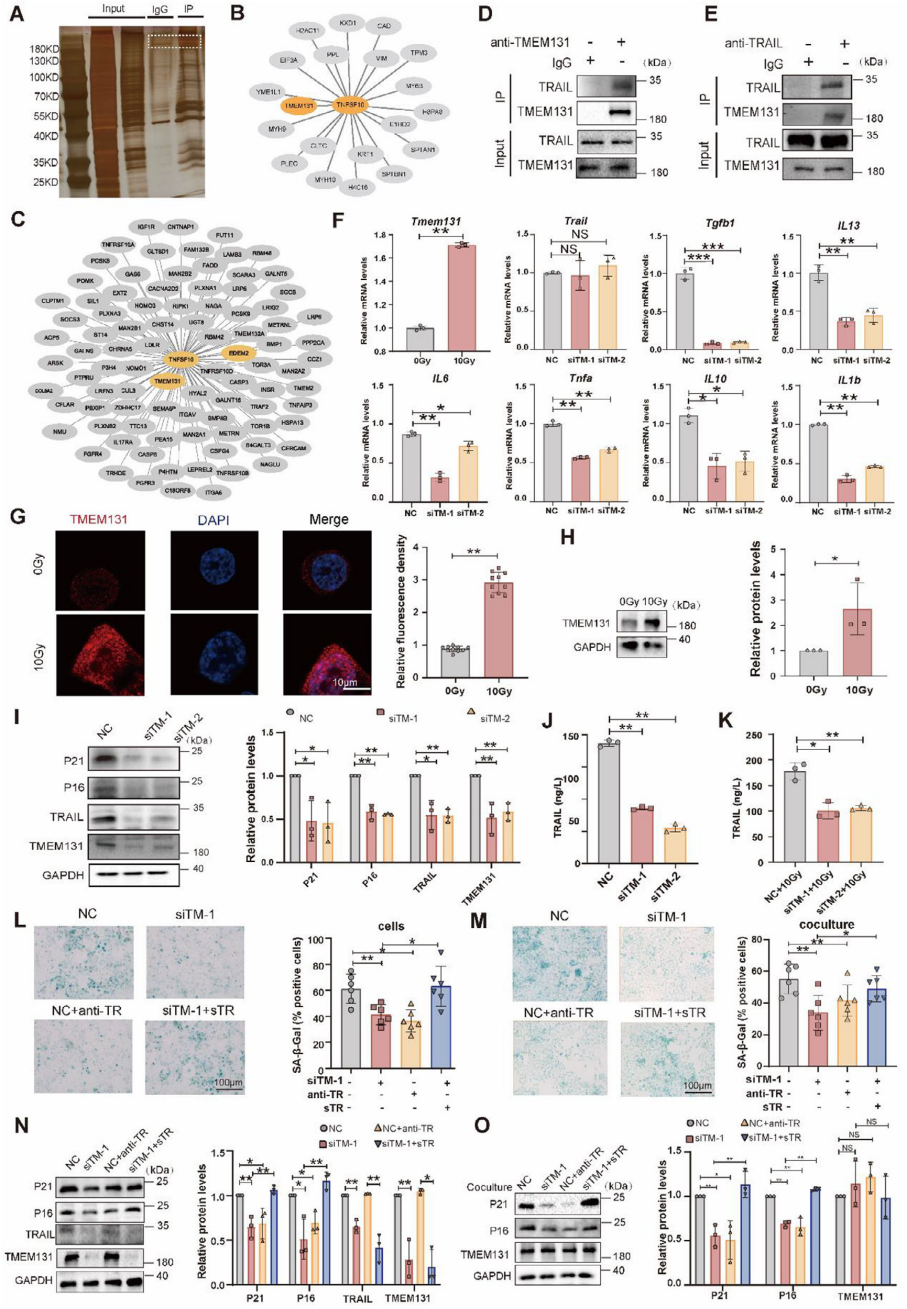
TMEM131 interacted with TRAIL and regulated its secretion and effects on cell senescence. A) TRAIL‐interacted proteins were detected by silver staining, emphasizing potential binding molecules. B,C) Identification of TMEM131 as an interactor of TRAIL using mass spectrometry B) and BioGrid database C). D,E) Co‐immunoprecipitation showing the interaction of TMEM131 with TRAIL. F) mRNA expression of TRAIL and cell senescence‐related cytokines in irradiated MLE‐12 cells with TMEM131 knockdown. G) Immunofluorescence of TMEM131 in MLE‐12 cells. H) Immunoblotting showing TMEM131 expression in MLE‐12 cells. I) P21, P16, TRAIL, and TMEM131 expression under TMEM131 knockdown in irradiated MLE‐12 cells. J,K) ELISA showing the expression of TRAIL in supernatant from irradiated MLE‐12 cells or unirradiated MLE‐12 cells under TMEM131 deficiency. L) β‐galactosidase staining with anti‐TR or sTR in irradiated MLE‐12 cells. M) β‐galactosidase staining with anti‐TR or sTR in unirradiated MLE‐12 cells cocultured with supernatant from irradiated MLE‐12 cells. N) Protein expression of cell senescence‐related molecules with anti‐TR or sTR in irradiated MLE‐12 cells using TMEM131 siRNA. O) Protein expression of cell senescence‐related molecules with anti‐TR or sTR in unirradiated MLE‐12 cells cocultured with supernatant from irradiated AECIIs. anti‐TR, TRAIL antibody; sTR, TRAIL recombinant protein; siTM‐1, siRNA‐1 for TMEM131; siTM‐2, siRNA‐2 for TMEM131. Data shown as mean ± SEM, N = 3–10; *p < 0.05; **p < 0.01; ***p < 0.001; NS, *p* ≥ 0.05.

Soluble TRAIL (sTRAIL) plays a significant role in anti‐tumor therapy, and targeted drugs based on sTRAIL or TRAIL receptors have been tested in clinical trials. However, the unsatisfied efficacy and safety concerns associated with existing drugs indicate that further research is still required for the development of TRAIL‐related therapeutics. As RILI involves neoplastic and healthy pulmonary tissues, and TRAIL can selectively induce tumor cell apoptosis while simultaneously suppressing the activation of autoreactive T cells, systemic inhibition of TRAIL or the use of TRAIL antibodies may trigger autoimmune disorders and accelerate tumor progression on cancer patients. Within this context, exploring the synthesis and secretion pathways of TRAIL may provide novel insights for research in RILI. TRAIL‐binding proteins were identified by immunoprecipitation mass spectrometry (**Figure** **4**A,B). Among the interacting proteins of TRAIL, TMEM131 was reported to be associated with the recruitment of COPII complex for transporting protein from ER to Golgi.^[^ ^50^ ^]^ TMEM131 was also predicted to interact with TRAIL in BioGrid website (Figure 4C). CO‐IP assays showed that TMEM131 interacted with TRAIL (Figure 4D,E). Moreover, purified glutathione S‐transferase (GST)‐fused TRAIL pulled down His‐TMEM131 in 293T cells, indicating the direct interaction between TRAIL and TMEM131 (Figure S6A, Supporting Information). The expression of TMEM131 was increased after irradiation (Figure 4F–H). To explore mechanism of radiation‐induced TMEM131 upregulation, we interrogated the KnockTF database (Version 2.0, http://www.licpathway.net/KnockTF/) for upstream transcription factors of TMEM131, and the results identified MYC, SRY‐box transcription factor 2 (SOX2), and GATA binding protein 3 (GATA3) as its potential transcriptional regulators (Figure S6B, Supporting Information). TMEM131 inhibition not only reduced senescence‐associated cytokine mRNA expression (Figure 4F) without alternation of TRAIL, but also decreased P21, P16 and TRAIL protein levels (Figure 4I), suggesting that TMEM131 regulated TRAIL at post‐transcriptional level. Flow cytometry and ELISA results revealed that TMEM131 knockdown downregulated TRAIL expression in the cell surface and supernatant (Figure 4J,K; Figure S6C, Supporting Information), suggesting TMEM131 modulated TRAIL distribution and trafficking. Rescue assays illustrated that TMEM131 promoted cell senescence via mediating TRAIL protein expression (Figure 4L–O). Collectively, these results indicated that TMEM131 participated in TRAIL‐mediated cell senescence, interacting with TRAIL and fostering its protein secretion.

### 2.5 TMEM131 Enhanced ER‐to‐Golgi Transport and Secretion of TRAIL

**Figure 5:**
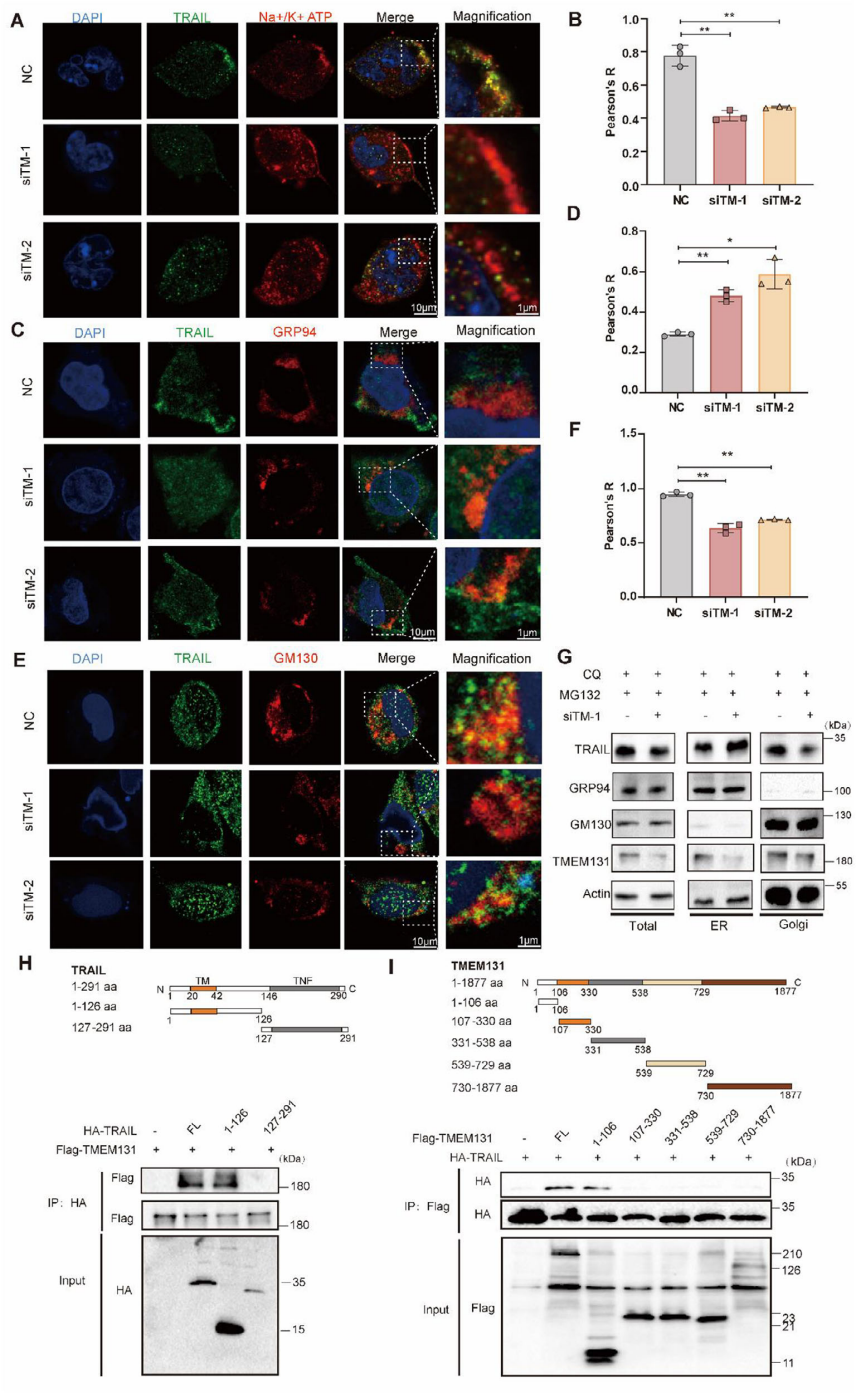
TMEM131 mediated intracellular transportation of TRAIL from ER to Golgi. A,B) Representative immunofluorescence images showing TRAIL localization on membrane after irradiation in TMEM131 knockdown MLE‐12 cells A) and the colocalization level between TRAIL and cell membrane (Na+/K+ ATP) B). C,D) Representative immunofluorescence images showing TRAIL localization on ER after irradiation in TMEM131‐deficient MLE‐12 cells C) and the colocalization level between TRAIL and ER (GRP94, D). E,F) Representative immunofluorescence images showing TRAIL localization on Golgi after irradiation in TMEM131‐deficient MLE‐12 cells E) and the colocalization level between TRAIL and Golgi (GM130, F). G) Immunoblotting displaying the effect of TMEM131 depletion on TRAIL protein distribution in ER and Golgi region. H) The interacting domains of TRAIL and TMEM131 were analyzed by using flag‐TMEM131 and full length or truncated HA‐TRAIL. I) The interacting domains of TMEM131 and TRAIL were analyzed by using HA‐TRAIL and full length or truncated flag‐TMEM131. siTM‐1, siRNA‐1 for TMEM131; siTM‐2, siRNA‐2 for TMEM131. Data shown as mean ± SEM, N = 3; *p < 0.05; **p < 0.01; NS, *p* ≥ 0.05.

ER and Golgi apparatus were critical for intercellular protein processing and protein secretion, and TMEM131 was reported to be involved in this process.^[^ ^50^, ^51^ ^]^ After silencing TMEM131, there was less colocalization of TRAIL and cell membrane marker Na^+^/K^+^ ATP (**Figure** **5A,B**; Figure S7A, Supporting Information). Interestingly, absence of TMEM131 increased distribution of TRAIL in ER (GRP94), while decreased the presence of TRAIL in Golgi (GM130, Figure 5C–F; Figure S7B,C, Supporting Information). Proteins were extracted from ER and Golgi region, respectively, and immunoblotting showed that TMEM131 depletion facilitated a remarkable accumulation of TRAIL in ER section and declined TRAIL expression in Golgi apparatus (Figure 5G). In addition, truncated plasmids were used to detect specific domains that TMEM131 interacted with TRAIL. Immunoblotting results demonstrated that amino acid domain (aa) 1‐106 of TMEM131 bound to aa 1‐126 of TRAIL (Figure 5H,I). The plasmids harboring full‐length TMEM131 and TMEM131 lacking 1‐106 aa (△106) were used to verify cellular functions of the specific domain. ELISA results revealed that 1‐106 aa deletion of TMEM131 downregulated TRAIL secretion from 293T and MLE‐12 cells (Figure S8A, Supporting Information). TMEM131 lacking 1‐106 aa also decreased senescent cell ratio and senescence protein expression (Figure S8B–D, Supporting Information). These findings suggested that TMEM131 provoked TRAIL trafficking from ER to Golgi, inhibiting which would interfere TRAIL protein maturation.

### 2.6 TMEM131 Assisted with COPII Molecule SEC23A Trafficking of TRAIL

**Figure 6:**
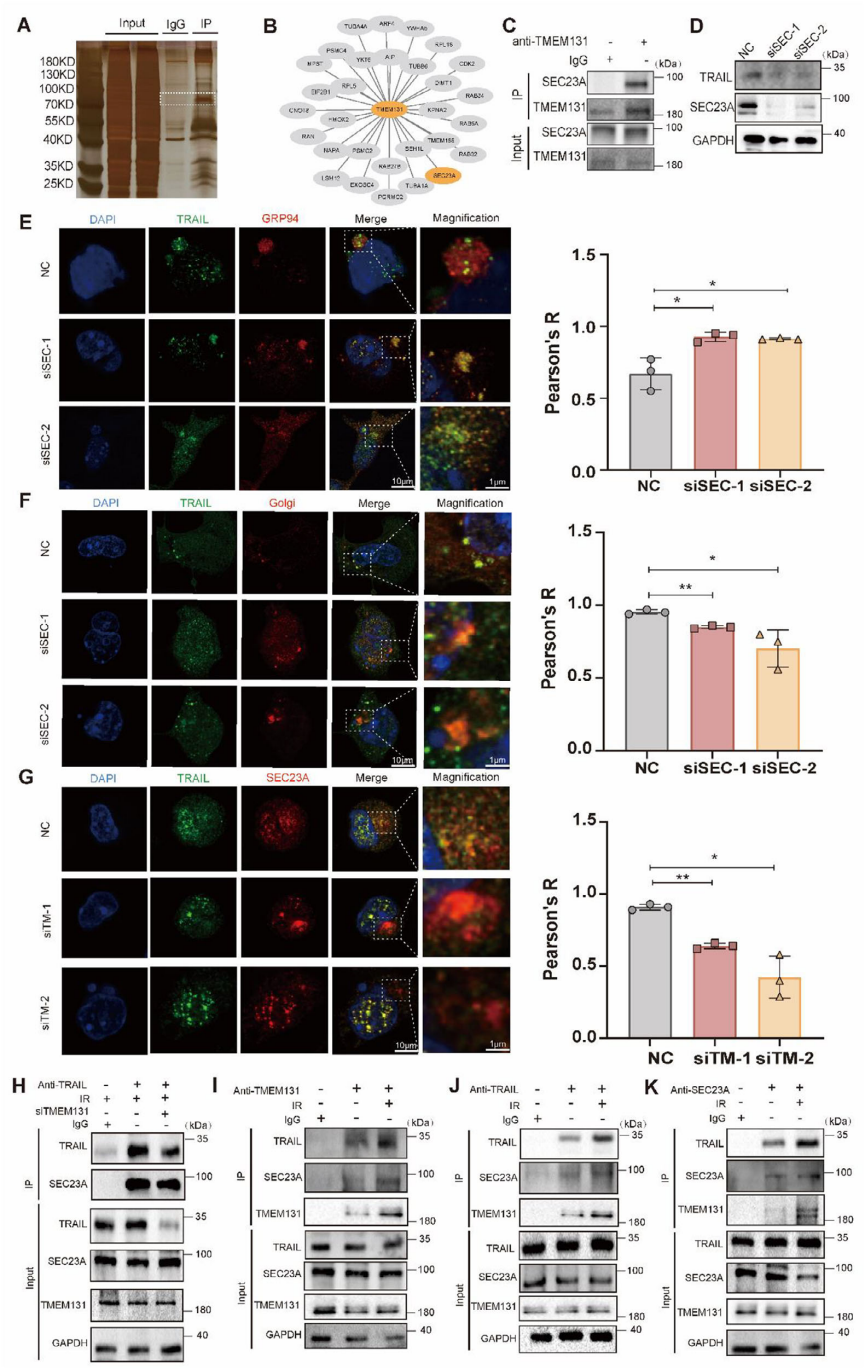
TMEM131 assisted COPII to traffick TRAIL from ER to Golgi. A) TMEM131‐interacted proteins were detected by silver staining, emphasizing potential binding molecules. B) Mass spectrometry analysis for MLE‐12 cells treated with anti‐IgG or TMEM131 antibody. C) Co‐immunoprecipitation showing the interaction of SEC23A with TMEM131. D) Immunoblotting of MLE‐12 cells with SEC23A knockdown. E,F) Representative immunofluorescence images showing TRAIL localization on ER (GRP94, E) and Golgi (GM130, F) after irradiation in SEC23A‐deficient MLE‐12 cells. G) Representative immunofluorescence images showing the colocalization between TRAIL and SEC23A under irradiation in TMEM131‐deficient MLE‐12 cells. H) The role of TMEM131 in interaction between TRAIL and SEC23A. I–K) The effect of irradiation in interaction among TMEM131, TRAIL and SEC23A. siTM‐1, siRNA‐1 for TMEM131; siTM‐2, siRNA‐2 for TMEM131; siSEC‐1, siRNA‐1 for SEC23A; siSEC‐2, siRNA‐2 for SEC23A. Data shown as mean ± SEM, N = 3; *p < 0.05; **p < 0.01; NS, *p* ≥ 0.05.

To further identify how TMEM131 assisted in transporting TRAIL to ER exit sites, we screened TMEM131 interactors through immunoprecipitation mass spectrometry. In eukaryotic cells, COPII vesicles are carriers for immature proteins trafficking from ER to Golgi.^[^ ^38^, ^52^ ^]^ We found SEC23A in precipitation conjugate (**Figure** **6**A–C), one of the COPII major proteins, which has been reported to interact with cargo molecules for delivering ER‐processed protein.^[^ ^39^, ^40^, ^53^, ^54^, ^55^ ^]^ Subsequent results showed that SEC23A deficiency reduced TRAIL expression, and increased the location of TRAIL in ER fraction rather than Golgi region (Figure 6D–F; Figure S9A,B, Supporting Information). To verify the role of TMEM131 in the regulation of SEC23A‐mediated TRAIL transport, we examined the interaction between SEC23A and TRAIL after TMEM131 knockdown. TMEM131 knockdown reduced the co‐localization of SEC23A and TRAIL, confirming the essential role of TMEM131 in enhancing the interaction between TRAIL and SEC23A (Figure 6G,H; Figure S9C, Supporting Information). Immunoprecipitation revealed that irradiation strengthened the association among TMEM131, TRAIL and SEC23A (Figure 6I–K). To sum up, TMEM131 served as a key bridge between TRAIL and SEC23A, promoting TRAIL trafficking from ER to Golgi.

### 2.7 Absence of TMEM131 Cause TRAIL Degradation via ERAD Mechanism

**Figure 7:**
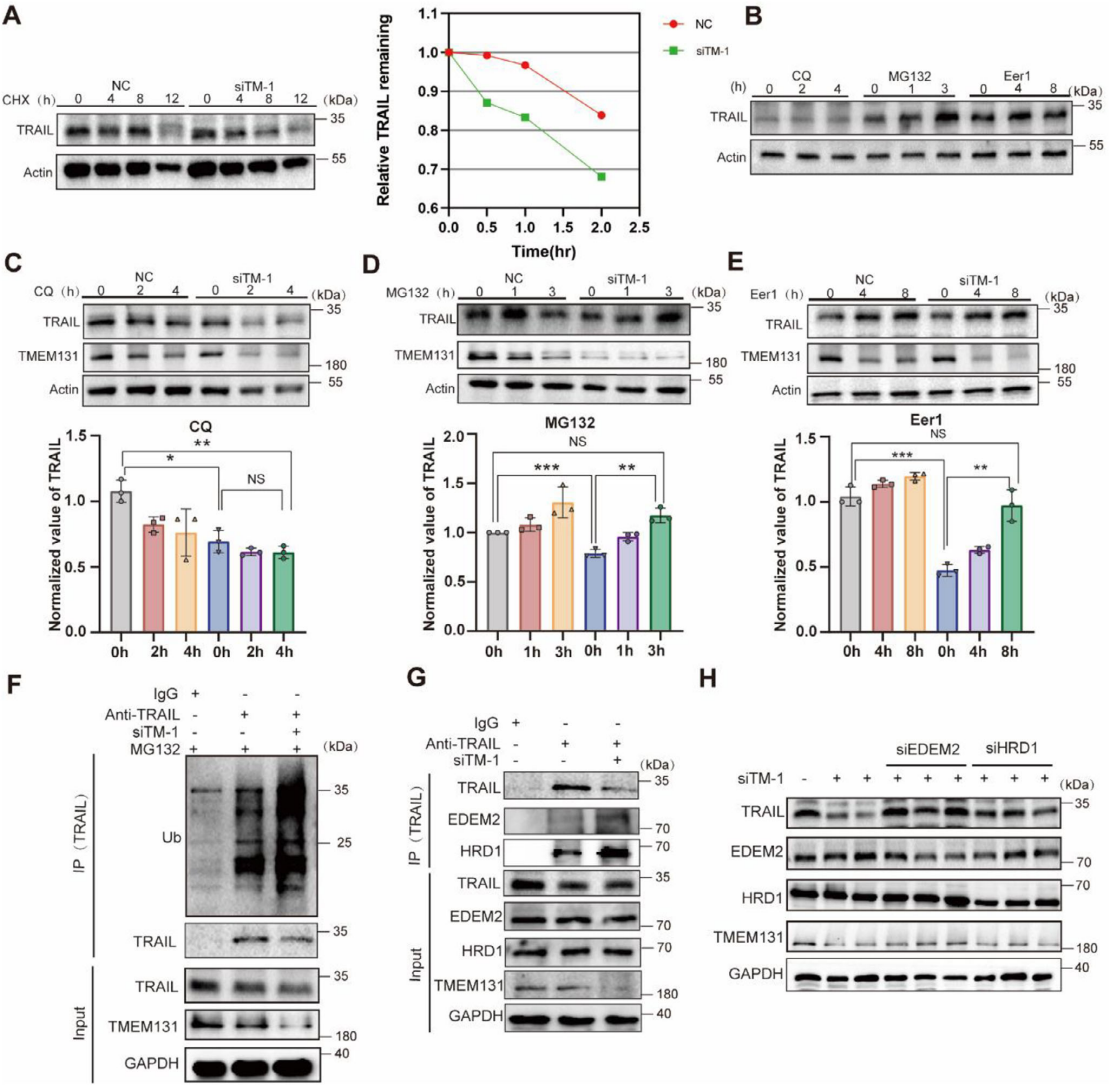
TMEM131 depletion induced ER‐associated degradation of TRAIL. A) Immunoblotting displaying the alternation of TRAIL protein in TMEM131‐deficient MLE‐12 cells treated with protein synthesis inhibitor (CHX, 50 µg mL^−1^). B) Immunoblotting showing the trend of TRAIL protein under autophagy inhibitor (CQ, 25 µM), proteasome inhibitor (MG132, 10 µM), ERAD inhibitor (Eer I, 10 µM). C–E) Immunoblotting displaying the alternation of TRAIL protein in TMEM131‐defiecent MLE‐12 cells treated with CQ, MG132, Eer I. F) TRAIL ubiquitination level after TMEM131 knockdown. G) Co‐immunoprecipitation representing the interaction of TRAIL with EDEM2 or HRD1 through ERAD pathway. H) Immunoblotting showing the expression of TRAIL transfected with TMEM131, EDEM2 and HRD1 siRNAs. siTM‐1, siRNA‐1 for TMEM131. Data shown as mean ± SEM, N = 3; *p < 0.05; **p < 0.01; ***p < 0.001; NS, *p* ≥ 0.05.

As TMEM131 knockdown led to TRAIL residency and accumulation in ER, we were curious about the fate and exit of these redundant proteins. ERQC monitor membrane proteins and secretion proteins in ER lumen. Abnormal proteins accumulation would trigger ERQC system, including ERAD, unfolded protein response and autophagy.^[^ ^43^, ^56^ ^]^ Utilizing protein synthesis inhibitor cycloheximide (CHX), we found that TMEM131 deficiency accelerated TRAIL degradation (**Figure** **7**A). Proteasomal inhibitor (MG132) or ERAD inhibitor eeyarestatin I (Eer I) mitigated TRAIL elimination rather than lysosome inhibitor CQ (Figure 7B), suggesting that TRAIL underwent ERAD‐mediated proteasomal degradation. TMEM131 depletion‐induced TRAIL decline could also be rescued by MG132 and Eer I (Figure 7C–E), suggesting that ERAD was involved in TMEM131‐mediated TRAIL degradation. TRAIL ubiquitination showed an increased trend upon TMEM131 downregulation (Figure 7F). ER degradation enhancing mannosidase (EDEM) 2, a key molecule in facilitating the presentation of misfolded glycosylated proteins for ERAD, was identified to interact with TRAIL in BioGrid database (Figure 4C). After demannosylated by EDEM2, proteins in ER lumen were captured by E3 ubiquitin ligase HRD1 complex for further ERAD pathway.^[^ ^57^, ^58^, ^59^, ^60^ ^]^ The interaction between TRAIL and EDEM2/HRD1 was significantly increased after TMEM131 deficiency (Figure 7G). Moreover, depletion of EDEM2/HRD1 considerably rescued the decrease of TRAIL expression mediated by TMEM131 silencing (Figure 7H). In summary, these results indicated that lack of TMEM131 triggered TRAIL‐ubiquitinated degradation through enhancing the recruitment to EDEM2/HRD1 complex by ERAD pathway.

### 2.8 Targeting TMEM131 in AECII Suppresses TRAIL Secretion and RILI in Vivo

**Figure 8:**
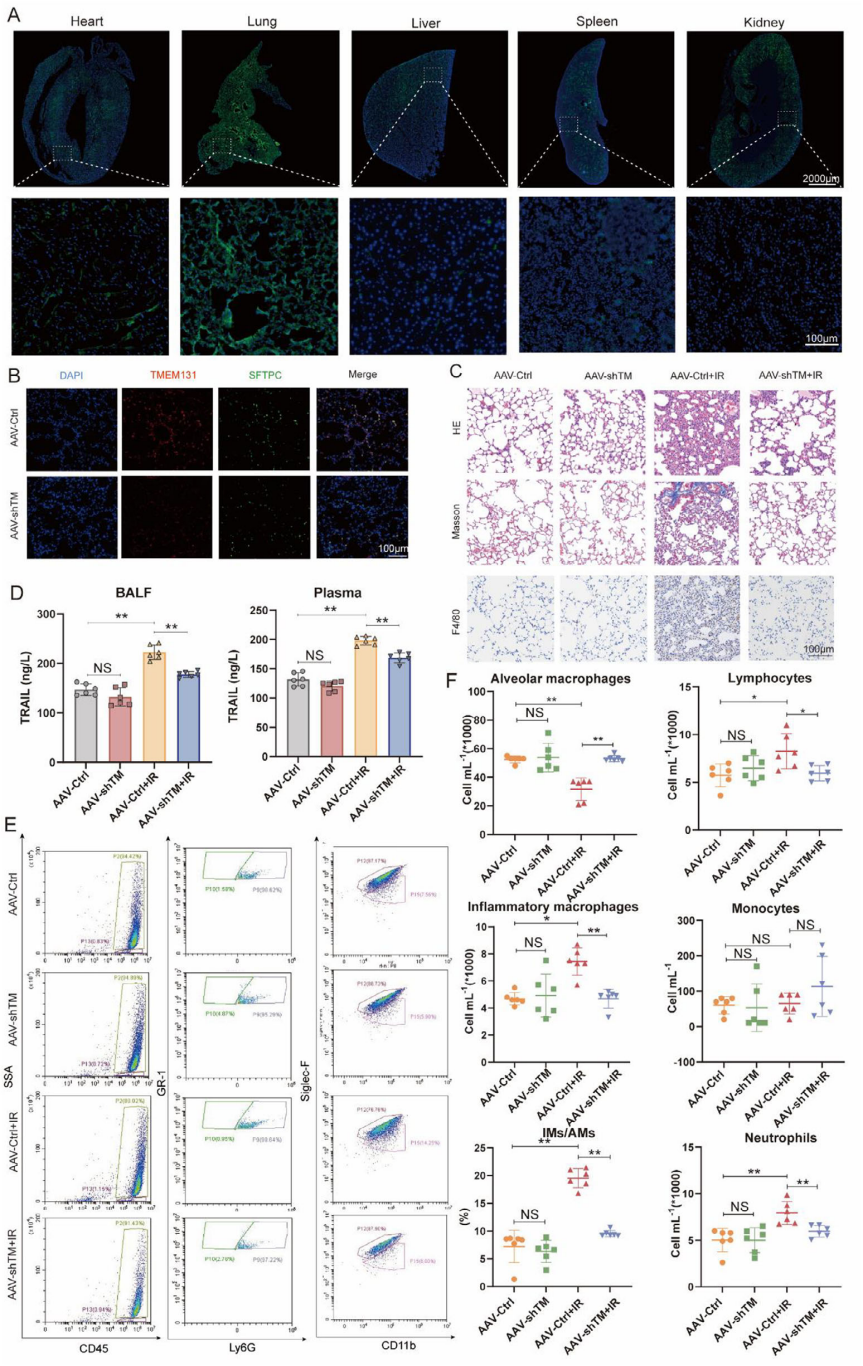
Targeted inhibition of TMEM131 in AECII suppressed TRAIL secretion and radiation‐induced lung injury in vivo. A) Autofluorescence images of heart, lung, liver, spleen and kidney following AAV6.2FF‐CMV‐EGFP infection. B) Immunofluorescence staining of TMEM131 and SFTPC in AAV‐Ctrl and AAV‐shTM lung tissues. C) Representative histological staining of normal and irradiated lung tissues from AAV‐Ctrl and AAV‐shTM mice. D) ELISA showing the expression of TRAIL in BALF (left) or plasma (right) from AAV‐Ctrl and AAV‐shTM mice. E,F) Representative flow cytometry analysis of BALF in AAV‐Ctrl, AAV‐shTM, AAV‐Ctrl +IR, AAV‐shTM+IR groups. Data shown as mean ± SEM, N = 6; *p < 0.05; **p < 0.01; NS, *p* > 0.05).

To assess the impact of TMEM131 on TRAIL secretion, mitophagy flux, and RILI progression in vivo, we utilized adeno‐associated virus (AAV) 6.2FF to construct AECII‐specific TMEM131 knockdown mice through intratracheal instillation (50 µL per mouse, with a viral titer of 1.5 × 10^11^ vg per animal). Considering flow cytometry analysis of BALF could be interfered with by EGFP autofluorescence, the AAV6.2FF‐SFTPC‐shTMEM131 (AAV‐shTM) and AAV6.2FF‐SFTPC‐Ctrl (AAV‐Ctrl) viruses were constructed without EGFP. AAV6.2FF‐CMV‐EGFP virus were used to verify the specificity and efficiency of AAV6.2FF on lung tissue infection. Two weeks after intratracheal instillation, autofluorescence images showed that AAV6.2FF‐CMV‐EGFP virus specifically and efficiently infected lung tissues in contrast with other organs (**Figure** **8**A). TMEM131 immunofluorescence intensity in AECIIs was significantly mitigated in AAV‐shTM group (Figure 8B), indicating the reduced expression of TMEM131 in AECIIs from the AAV‐shTM group.

RILI models were next established in AAV‐shTM and AAV‐Ctrl mice, which underwent a single 20 Gy thoracic irradiation. Immunohistochemistry revealed that lung tissues from the AAV‐shTM RILI group exhibited less consolidation, along with significantly lower degrees of fibrosis and macrophage infiltration compared to the AAV‐Ctrl RILI group (Figure 8C). ELISA results demonstrated that the AAV‐shTM group significantly downregulated the expression levels of sTRAIL in both BALF and plasma (Figure 8D). Flow cytometry of BALF showed a significant reduction in the numbers of pro‐inflammatory macrophages, lymphocytes, and neutrophils in the AAV‐shTM RILI group (Figure 8E,F). Transmission electron microscopy illustrated a significant increase in the number of mitophagosomes in AECIIs from the AAV‐shTM RILI group (Figure S10A,B, Supporting Information). Moreover, the fluorescent intensity of PARKIN, LC3B in SFTPC‐positive cells were highly elevated in the AAV‐shTM RILI group, while the intensity of TRAIL was reduced (Figure S10C,D, Supporting Information). These results confirmed that AECII‐specific TMEM131 inhibition in vivo decreased TRAIL secretion, increased mitophagy influx, and suppresses the progression of RILI.

## 3 Discussion

RILI, as one of the major complications of radiotherapy, irreversibly deteriorates life quality and prognosis of thoracic cancer patients. Given the inefficiency and toxicity of current RILI treatments, there is an urgent need to identify reliable radioprotectors.^[^ ^61^, ^62^, ^63^ ^]^ Importantly, accumulating evidence revealed that eliminating senescent cells in RILI mice using senolytics drugs reversed lung fibrosis.^[^ ^64^ ^]^ Moreover, AECII senescence, rather than apoptosis, was positively correlated with irradiation dose,^[^ ^14^ ^]^ thereby targeting AECII senescence might be a promising radioprotection strategy for RILI.

In our study, cell senescence was identified as an important behavior during RILI development by RNA transcriptome sequence in the RILI mice model. Consistent with previous studies, immunohistochemistry and flow cytometry revealed that administration of the senolytics agent DQ remarkably alleviated lung fibrosis. Through single‐cell transcriptomics and multi‐color immunofluorescence, AECIIs were identified as the primary cells undergoing senescence, which was consistent with previous research.^[^ ^19^ ^]^ Irradiated AECIIs secreted TRAIL at high levels, as evidenced by supernatant mass spectrometry and immunofluorescence. Moreover, ELISA of plasma samples revealed that TRAIL expression was significantly higher in RILI patients. Subsequent lung injury in *Trail*‐cKO mice treated with DQ was significantly decreased with less lung consolidation and immune cell infiltration. Enrichment analysis showed that TRAIL ablation in AECIIs influenced cell senescence and mitophagy. Our results also revealed that TRAIL knockdown or anti‐TRAIL treatment reduced the expression of AECII senescent cytokines and proteins. Moreover, reduced SASP effect from TRAIL‐deficient cells decreased the conversion of fibroblasts to myofibroblasts. These results elucidated that blocking TRAIL in senescent AECIIs would be of great importance in inducing RILI.

The enrichment analysis in RILI lung RNA transcriptome sequence and elevated mitochondrion membrane potential suggested that mitophagy might be implicated in TRAIL‐mediated cell senescence. Reactive oxygen stress or cell senescence stimuli activate mitophagy, which means that damaged mitochondria would be sequestered into autophagosomes and fused with lysosomes for lysosomal degradation. Defective mitophagy results in the accumulation of damaged mitochondria, thereby exacerbating cellular dysfunction and accelerating senescence in turn.^[^ ^65^, ^66^, ^67^ ^]^ Meanwhile, the role of TRAIL in autophagy is multifaced. In multiple cancer cell lines, TRAIL increases autophagy influx rather than apoptosis.^[^ ^68^, ^69^, ^70^ ^]^ However, TRAIL induces Beclin1 protein cleavage or influences P62 expression, ultimately blocking autophagy in other cancer cell lines.^[^ ^71^, ^72^, ^73^, ^74^ ^]^ The mechanism by which TRAIL influences mitophagy remains unclear. Formation, extension, maturation and termination of autophagosome comprise the autophagy process,^[^ ^67^ ^]^ with mTOR signaling pathway acting as a negative regulator.^[^ ^75^ ^]^ Additionally, mTOR inhibits transcriptional regulation of lysosomal biogenesis, which prevents autophagosome‐lysosome fusion.^[^ ^76^ ^]^ TRAIL knockdown resulted in a tendency for mitophagy autophagosome, autophagolysosome and mitophagy flux. Moreover, TRAIL triggered mTOR pathway activation, thus inducing cell senescence. This explains the enhanced anti‐senescence efficacy of combining TRAIL knockout and DQ in RILI mouse model. TRAIL ablation prevents the initiation of cellular senescence in AECIIs, while DQ eliminates pre‐existing senescent cells.^[^ ^77^ ^]^ This combination presents a promising novel therapeutic paradigm for RILI. Our findings strongly indicated that TRAIL facilitated cell senescence via mTOR‐inhibited mitophagy pathway.

Our results indicated that TRAIL activated AECII senescence and mTOR pathway, and that DR5 knockdown alleviated cellular senescence. Previous studies revealed that TRAIL induces PI3K/AKT/mTOR signaling pathway through DR5,^[^ ^78^ ^]^ and that activated PI3K/AKT/mTOR pathway is associated with P21/P16 upregulation and cellular senescence.^[^ ^79^ ^]^ Therefore, we believe that TRAIL‐DR5 interaction initiate AECII senescence and P21/P16 upregulation through PI3K/AKT/mTOR pathway.

Despite extensive studies on the role of sTRAIL in cancer and inflammation, the regulatory mechanisms underlying sTRAIL secretion remain elusive. Targeting TRAIL to specific senescence cells and exploring the mechanism of sTRAIL secretion would be essential. Most proteins undergo complicated synthesis processes. Nascent peptides are guided to rough ER, followed by processing and folding. COPII vesicles envelop immature proteins from ER membrane to Golgi apparatus for further packaging.^[^ ^80^ ^]^ TMEM131 is predicted to be a biomarker in idiopathic pulmonary hypertension^[^ ^81^ ^]^ and colorectal cancer.^[^ ^82^ ^]^ In *Caenorhabditis Elegans*, TMEM131 recruits COPII complex to facilitate the transport of extracellular matrix proteins such as collagen 19 from ER to Golgi apparatus, thereby enhancing its secretion.^[^ ^50^, ^51^ ^]^ The function of TMEM131 in RILI remains unclear. SEC23A was reported to identify cargo proteins in ER lumen and transfer them to COPII vesicles.^[^ ^83^, ^84^ ^]^ Moreover, SEC23A production is directly correlated with the efficiency of protein transport. SEC23A enhances the secretion of S100A8, platelet factor‐4 in melanoma,^[^ ^85^, ^86^ ^]^ promotes tubulointerstitial nephritis antigen like 1 and insulin like growth factor binding protein 4 secretion in cancer metastasis,^[^ ^87^ ^]^ and augments the level of sterol regulatory element‐binding protein 1 in hepatic lipid.^[^ ^41^ ^]^ Responsible for translocation of most secretory proteins from ER to Golgi, SEC23A is an essential modulator in proteomics. In this work, TMEM131 was found to be a TRAIL‐interacting protein, which also mediated cell senescence via regulating TRAIL secretion. SEC23A was identified to mediate TRAIL binding to COPII complex, which was bridged by TMEM131. The absence of TMEM131 disrupted the binding between TRAIL and SEC23A, contributing to the aberrant accumulation of TRAIL in ER and declined presence in Golgi. The role of TMEM131 and SEC23A in regulating TRAIL secretion shed novel insights into diseases targeting TRAIL.

MYC, SOX2, and GATA3 were potential modulator of TMEM131 transcription, and reported to be upregulated by irradiation. The expression of MYC were upregulated in radiation‐induced kidney injury.^[^ ^89^ ^]^ SOX2 regulates radiosensitivity in pancreatic cancer and shows upregulated expression in tumor organoids after 8 Gy irradiation.^[^ ^90^ ^]^ GATA3 is upregulated following radiotherapy for benign prostate conditions.^[^ ^91^ ^]^ We hypothesize that these potential transcription factors with high expression level might be responsible for the upregulation of TMEM131 expression post‐radiotherapy.

After silencing TMEM131, immature TRAIL would be sequestered in ER. To ensure cellular protein homeostasis, ERQC would be activated by TRAIL retention in ER. ERQC verifies the exit of properly addressed proteins by COPII complex while traps abnormal proteins in ER and subsequently causes protein degradation.^[^ ^58^, ^59^ ^]^ EDEM and HRD1 complex are key modulators in ERAD. Glycoprotein‐related ERAD was reported to be initiated by EDEM2.^[^ ^88^ ^]^ After administration of corresponding degradation inhibitors, we found that TMEM131 absence‐induced TRAIL retention in ER would be degraded by ERAD pathway. We also found that the interaction among EDEM2, HRD1 and TRAIL was increased after silencing TMEM131. Depletion of EDEM2 and HRD1 rescued TRAIL expression after TMEM131 deficiency. These findings suggested that EDEM2 and HRD1 complex mediated TRAIL ubiquitination and degradation in ERAD pathway.

**Figure 9:**
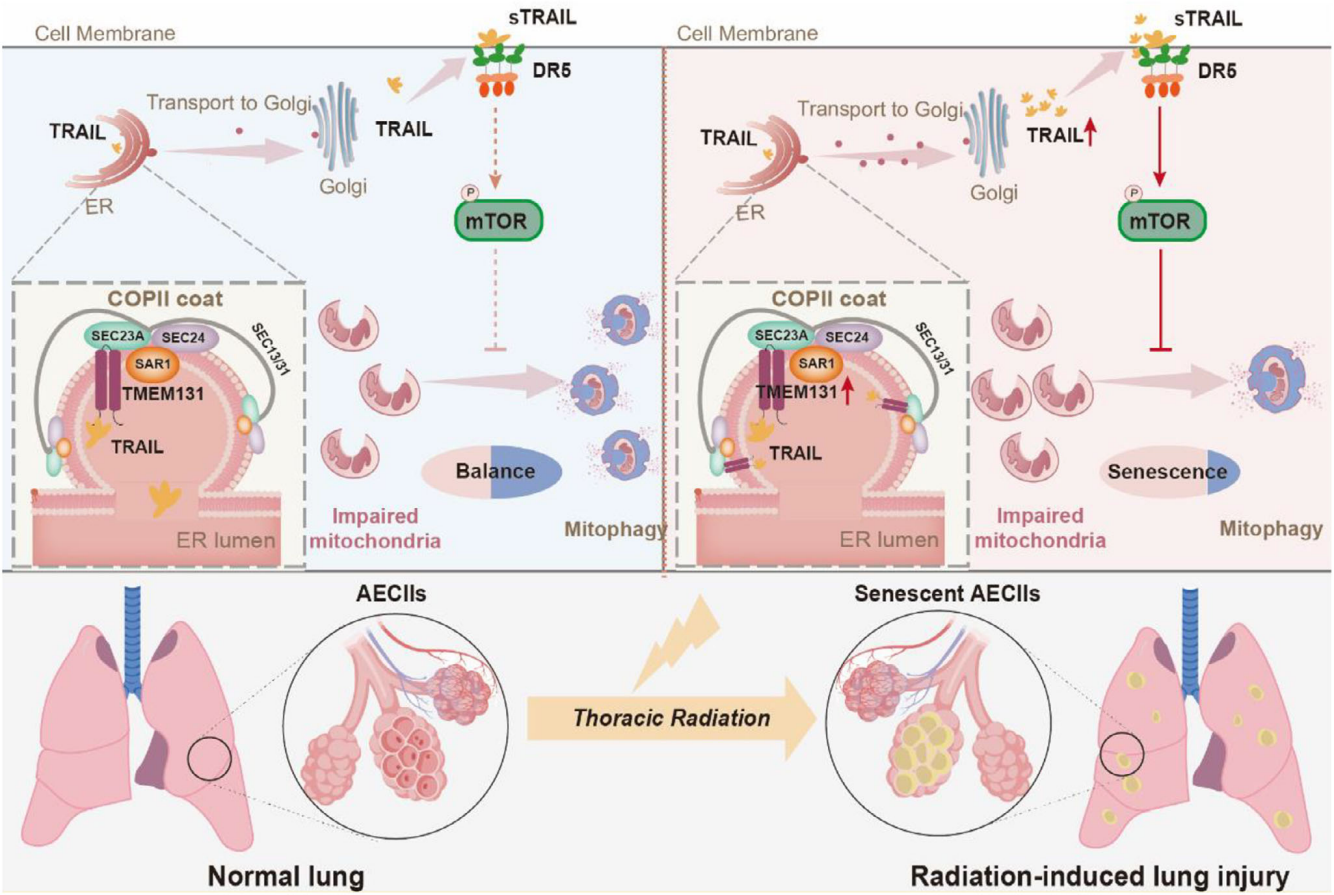
Mechanism diagram. TMEM131 in AECIIs was upregulated during radiation, which promoted the binding between TRAIL and COPII molecule SEC23A. This interaction subsequently facilitated the transport of TRAIL from endoplasmic reticulum to Golgi, thereby boosting TRAIL secretion. sTRAIL combined with DR5 receptor on AECIIs to activate mTOR pathway, ultimately inhibiting mitophagy flux. Accumulation of impaired mitochondria lead to AECII senescence in the radiated area, and ultimately exacerbated post‐IR lung dysfunction (depicted on the right in irradiated AECIIs).

TRAIL affects RILI via regulating AECII senescence through mTOR‐mitophagy pathways, and targeting the upstream proteins involved in TRAIL secretion can achieve a more effective role while avoiding influencing anti‐tumor effect of TRAIL from immune cells. In summary, our study illuminated the mechanisms underlying TRAIL‐regulated cell senescence and TRAIL maturation. We discovered that TRAIL, through its mediation of mTOR‐dependent cell mitophagy, played a pivotal role in radiation‐induced cell senescence. Additionally, targeting TMEM131 reduced interaction of TRAIL and COPII component SEC23A, decreasing TRAIL secretion (**Figure** **9**). Aberrantly accumulated TRAIL in ER by depletion of TMEM131 was subsequently degraded by ERAD pathway. This work unveils the role of TRAIL in cell senescence and pinpoints a novel strategy for controlling TRAIL secretion in curing RILI.

## 4 Experimental Section

### Patients

Lung cancer patients (≥18 years) receiving radiotherapy at the Department of Pulmonary Oncology, Zhongnan Hospital of Wuhan University from February 2023 to August 2024 were enrolled. Inclusion criteria included: 1) patients received radiotherapy; 2) patients met the diagnostic criteria for acute RILI after radiotherapy; 3) patients had a Karnofsky Performance Status score >60 with expected survival time more than 3 months. Exclusion criteria included: 1) severe impairment of heart, lung, liver, and kidney functions and severe blood infectious diseases; 2) RILI before radiotherapy; 3) lack of biomedical information and clinical samples. Of the 14 patients screened for inclusion, 1 patient had acquired immune deficiency syndrome and 4 patients lack plasma samples at the collection time point. Therefore, a total of 9 patients with paired plasma samples (Pre‐RT and RILI) were included. Clinical characteristics of patients are presented in Table S1 (Supporting Information). The research utilizing human plasma tissues have received informed consent from patients and approval from the Ethics Committee of Zhongnan Hospital of Wuhan University (2024092K).

### Animals

Animal procedures were authorized by the Animal Care Committee of Wuhan university (ZN2023244). Newborn Sprague‐Dawley rats (1–2 days) were purchased from Hubei Provincial Center for Disease Control and Prevention. *Trail^flox/flox^* mice and *Sftpc‐CreER^T2^* mice (C57BL/6) were purchased from Biocytogen. Mice were housed in the A3 Laboratory Animal Research Center of Wuhan University. The inducible AECII‐specific TRAIL conditional knockout (*Sftpc‐CreER^T2+^/Trail^flox/flox^*) mice (4 weeks) were administered intraperitoneally with tamoxifen at 0.15 mg g^−1^ daily for 5 consecutive days, and their respective control (*Sftpc‐CreER^T2−^/Trail^flox/flox^*) mice received the same volume of corn oil. The chest of mice was irradiated by the small animal radiation research platform (PXI X‐RAD 225Cx, Gulmay, CT, USA) at 20 Gy. Dasatinib (1 mg/kg) and quercetin (10 mg kg^−1^) were delivered by intraperitoneal injection once a week. After 6 weeks, all animals were euthanized. Primer sets were used: *TRAIL^flox/flox^*, 5′‐TGCAGAGTAGAAACAATGCCATTAGG‐3′ and 5′‐GTCGTTCTTATTTTCCCTCATATGCAC‐3′; *Sftpc‐CreER^T2^*, 5′‐TTGGCCTTGGCTGAGCTTAGACAT‐3′ and 5′‐CCTTCACGACATTCAACAGACCTT‐3′.

To generate AECII‐specific TMEM131 knockdown model, C57BL/6 mice (5 weeks) were treated with AAV6.2FF virus via intratracheal instillation (50 µL per mouse, with a viral titer of 1.5 × 10^11^ vg per animal) followed by 2 weeks interval. All animals were sacrificed at 6 weeks after 20 Gy thoracic irradiation administration.

### Cells

Mouse alveolar epithelium MLE‐12 cells were obtained from FengHui Biological Company. Human embryonic kidney (HEK) 293T cell was purchased from the Type Culture Collection of the Chinese Academy of Science. Primary newborn alveolar epithelium cells were isolated from lungs of newborn rats within 2 days. Lungs from rats with 75% alcohol disinfection were obtained and completely rinsed in phosphate buffer saline (PBS). The lung tissue was then cut into 1‐mm size in dulbecco's modified eagle medium (DMEM) supplemented with 10% fetal bovine serum (FBS) and 1% penicillin‐streptomycin. Subsequently, these isolated tissues were transferred to a container full of mixed enzyme solution (0.25% trypsin and 0.1% collagenase) in shaker incubator at 37 °C for 20 min. The digestion supernatant was collected in another tube with DMEM (50% FBS) to halt the digestion. After repeated digestion for 6–8 times, the collected solution was filtered through 70‐µm mesh filter and centrifuged at 130 *g* for 8 min to remove AT1 cells. Cell pellets were resuspended in DMEM and plated in 10‐cm culture dishes coated with CD45 and CD32 antibodies for 2 h. The adherent cells were lung fibroblasts. Cell precipitates were further lysed with red blood cell lysis buffer at room temperature for 3 min and then neutralized with DMEM. After centrifugation at 130 *g* for 8 min, cell pellets were collected and cultured in DMEM with 10% FBS and maintained in a 37 °C incubator with 5% CO_2_.^[^ ^92^, ^93^ ^]^

### Reagents

Reagents including dasatinib (CDS023389, Sigma), quercetin (Q4951, Sigma), AAV6.2FF virus (HANBIO Biotechnology), recombinant mouse TRAIL (315‐19, Peprotech), anti‐TRAIL antibody (14‐5951‐85, eBioscience), JC‐1(40705ES03, YEASEN Biotech), mcherry‐enhanced green fluorescent protein (EGFP)‐LC3B adenovirus (HANBIO), mtKeima adenovirus (HANBIO), Bafilomycin A1(A8510, Solarbio), Mdivi‐1 (S7162, Selleck), Silver staining (BL620A, Biosharp), Minute endoplasmic reticulum enrichment Kit (ER‐036, Invent), Minute Golgi section enrichment Kit (GO‐037, Invent), MG132 (S2619, Selleck), chloroquine (CQ, C6628, Sigma‐Aldrich) and Eer I (10 012 609, Cayman) were purchased from the corresponding suppliers.

### Flow Cytometry

Cells from bronchoalveolar fluid were collected by centrifugation at 800 *g* 4 °C for 10 min, and then were lysed with red blood cell lysis buffer at room temperature for 3 min. After washed with PBS, cell pellets were stained with CD45 APC (103 111, Biolegend), Ly‐6G FITC (127 605, Biolegend), Gr‐1 PE/Cy7 (108 415, Biolegend), CD11b Pacific Blue (101 223, Biolegend), CD11c Percp/cy5.5 (117 316, Biolegend) anti‐mouse bodies and Siglec‐F PE‐CF594 (562 757, BD) in dark at 4 °C for 30 min. Flow cytometry was performed using Beckman Cytoflex. Gating strategies were shown in Figure S1 (Supporting Information). Cells were washed with PBS and resuspended with corresponding buffer. For apoptosis, cells were incubated with Annexin V‐FITC for 30 min and PI for 5 min following the instructions of Apoptosis Kit (BestBio, China). For mitochondrial membrane potential staining, cells were treated with mitochondrial membrane potential fluorescent probe (40705ES, Yeasen) at 37 °C for 20 min. For detection of TRAIL expression level on cell membrane, cells were stained with anti‐TRAIL antibody (50‐133‐51, eBioscience) in dark at 4 °C for 30 min. After washing with PBS, single cell suspension was analyzed by flow cytometry (Beckman Cytoflex).

### RNA Interference

Cells were transfected with siRNAs targeting TRAIL or TMEM131 or mouse TRAIL effectors using lipofectamine 3000 transfection reagent (Invitrogen), according to manufacturer's protocol. For rescue experiments, cells were transfected with siRNAs for 24 h, followed by 10 Gy irradiation and treatment with TRAIL antibody (2 µg/ml) or TRAIL recombinant protein (200 ng mL^−1^) for 48 h. The siRNA sequences were displayed in Table S2 (Supporting Information).

### ER and Golgi Protein Extraction

By utilizing the characteristics of organelles with varying sizes and densities to sediment at different rates under centrifugal force in density media (iodixanol aqueous solution or sucrose solution), ER and Golgi protein are separated and purified through different centrifugal force (according to the instructions of ER‐036 and GO‐037 from Invent company).

### Protein Purification

pT7‐GST, pT7‐GST‐TRAIL or pT7‐His‐TMEM131 plasmid was transformed into Escherichia coli strain BL21 competent cells and proteins were induced with IPTG at 16 °C for 12 h. Bacteria were harvested by centrifugation and lysed by ultrasonication. Lysate was subjected to glutathione‐agarose beads or Ni‐NTA beads (Yeasen) following the manufacturer's protocols; The pull‐down assays of proteins followed the same steps of *Immunoprecipitation* section.

### Immunoblotting and Immunoprecipitation

Protein extraction was obtained using RIPA lysis buffer (Beyotime) with phosphatase and protease inhibitors. After centrifuged at 12 000 rpm for 10 min, supernatant from lysate were collected and then boiled with 5 × loading buffer. Protein samples were used for immunoblotting. Cells were incubated in immunoprecipitation (IP) lysis buffer. After centrifuged at 4 °C 12 000 rpm for 10 min, supernatants were treated with protein A/G agarose beads (sc‐2003, Santa Cruz) and antibodies at 4 °C overnight. Subsequently, the combination of immunoprecipitated‐beads was repeatedly washed using NaCl buffer. Beads were boiled with 2 × loading buffer, which were protein samples for IP analysis. Information of antibodies were presented in Table S3 (Supporting Information).

### Mass Spectrometry

For cell precipitation, proteins that interact with TRAIL or TMEM131 were collected from MLE‐12 cells. Cell lysates were immunoprecipitated with a specific antibody (for TRAIL or TMEM131) or isotype antibody with protein A/G beads (MCE, HY‐K0202) at 4 °C overnight on a rotator. Following 5 washes with NP‐40 lysis buffer, the eluted samples were gathered and subjected to tandem mass spectrometry (LC‐MS/MS) on Orbitrap Fusion Lumos mass spectrometer (Thermo Fisher). The LC‐MS/MS data were analyzed by Mascot (v2.3.02). For cell supernatant, cell medium was harvested from MLE‐12 cells after centrifugated twice at 1500 *g* for 10 min. Proteins in the supernatants were lysated with SDT (4% Sodium dodecyl sulfate (SDS), 100 mM Dithiothreitol, 100 mM Tris‐HCl, pH7.6). After boiled for 5 min, proteins underwent SDS‐ polyacrylamide gel electrophoresis (PAGE) electrophoresis. Samples were then digested with trypsin by filter aided proteome preparation method. Following by desalted with C18 Cartridges, vacuum centrifugation and reconstituted in 40 µL of 0.1% (v/v) formic acid, the peptides from each sample were analyzed by Orbitrap Astral mass spectrometer (Thermo Scientific). Analysis of DIA data was carried out by DIA‐NN 1.8.1.

### Quantitative Real‐Time PCR (qRT‐PCR)

Cells and tissues were lysed by TRIzol (Vazyme) to collect RNA samples, which were reverse transcribed into cDNA by HiScript QRT SuperMix kit (Vazyme). Quantitative PCR (qPCR) were carried out using ChamQTM SYBR qPCR Master Mix (Vazyme) on Bio‐Rad CFX96. The primer sequences were listed in Table S4 (Supporting Information).

### Immunofluorescence

Cells were seeded on coverslips and then were fixed with 4% paraformaldehyde at room temperature for 15 min. Next, samples were permeabilized using 0.2% Triton‐100 at room temperature for 15 min, and blocked in 5% bovine serum albumin (BSA) at room temperature for 1 h. After incubated with primary antibodies at 4 °C overnight, coverslips were treated with indicated secondary antibodies. Coverslips were placed on glass slides using a solution containing DAPI. Images were acquired by fluorescence microscope (MSHOT) and confocal fluorescence microscopy (Leica).

### Immunohistochemistry

Tissues from mice were fixed with 4% paraformaldehyde and embedded using paraffin. Following by deparaffinization, rehydration, wax block, the slides were incubated using particular primary antibodies and secondary antibodies. Images were obtained by light microscopy (Olympus).

### Enzyme‐Linked Immunosorbent Assay (ELISA)

Culture medium from MLE‐12 cells was collected 48 h after irradiation. The concentration of TRAIL was measured by TRAIL ELISA kit (MM‐47266M1, MEIMIAN). Plasma samples from 9 patients were harvested at 2 time points (before receiving radiotherapy and after diagnosed with RILI). After centrifuged at 3000 rpm for 20 min, TRAIL expression was analyzed by TRAIL ELISA kit (MM‐1938H1, MEIMIAN).

### Bioinformatics

Single‐cell transcriptomics data of lung tissues with irradiation or not were collected from Gene Expression Omnibus (GEO, GSE206426, http://www.ncbi.nlm.nih.gov/geo). Protein interactions were revalidated in BioGrid database (https://thebiogrid.org).

### Statistical Analysis

All statistical analyses were performed in GraphPad Prism (version: 8.0.2), with data presented as mean ± standard deviation (SD). For paired data from two samples, paired Wilcoxon signed‐rank tests were used for non‐normally distributed data. For unpaired data from two samples, Student's *t*‐test (for data with equal variances) or Welch's *t*‐test (for data with unequal variances) was employed for normally distributed data, whereas non‐parametric tests were utilized for non‐normally distributed data. *p* < 0.05 was defined as statistically significance.

## Conflict of Interest

The authors declare no conflict of interest.

## Author Contributions

L.H., C.W., and X.G. contributed equally to this work. L.H., C.W., and X.G. designed the experiment; L.H., C.W., Y.L., and J.Z. performed the experiments; L.H., F.H., Z.Z., and J.L. analyzed data; W.O. collected the clinical samples; L.H. wrote the manuscript; L.H., Y.G., and C.X. confirmed the raw data and revised the manuscript.

## Supporting information



Supporting Information

## Data Availability

The data that support the findings of this study are available from the corresponding author upon reasonable request.
