# Supplementary material for: TMEM131‐Mediated Soluble TRAIL Triggered Type II Alveolar Epithelial Cell Senescence in Radiation‐Induced Lung Injury
Source: Adv Sci (Weinh). 2025 Nov 26;13(6):e09973. doi: 10.1002/advs.202509973 (PMC12866828; doi:10.1002/advs.202509973)
Supplement: Supplementary file 1 — Supporting Information [file ADVS-13-e09973-s001.docx]

**
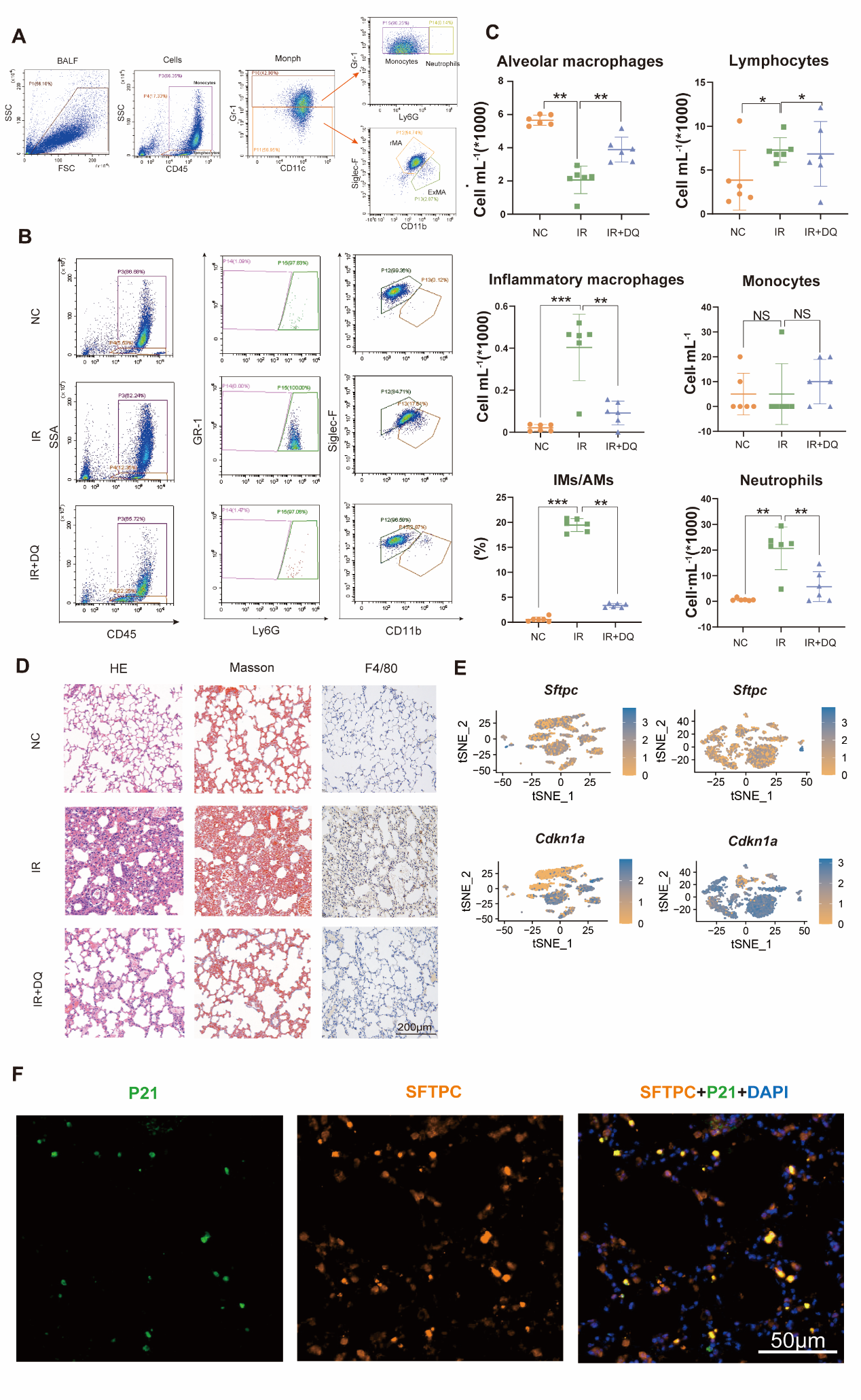
Figure S1.** Cell senescence of AECIIs is a main source of RILI. A) Gating strategy for flow cytometry of BALF cells. Lymphocytes were sorted in accordance with low SSC and low FSC in CD45+ cluster. From mononuclear phagocytes (MonPh, high SSC in CD45 cluster), alveolar macrophages (CD11binterSiglec-Fhigh) and inflammatory macrophages (CD11bhighSiglec-Flow) were gated in CD11chighGR-1low population, and neutrophils (GR-1+Ly-6Ghigh) and monocytes (GR-1+Ly-6G-) were gated in CD11chighGR-1high population. B) Flow cytometry for infiltrated immune cells in BALF. C) Quantification of flow cytometry for macrophages, monocytes and neutrophils in BALF. D) Representative histochemistry with H&E, Masson and F4/80 staining of mouse lung tissues. E) Single-cell transcriptomic analysis illustrated that cell senescence marker cyclin-dependent kinase inhibitor 1A (*Cdkn1a*, P21) was co-localized with AECIIs marker (*Sftpc*) in the RILI mouse lung tissues. F) Immunofluorescence of P21 protein co-localization with AECIIs (SFTPC). IMs/AMs, Inflammatory Macrophages/Alveolar Macrophages; Data shown as mean ± SEM, N = 6; *, *P* < 0.05; **, *P* < 0.01; ***, *P* < 0.001; NS, *P* > 0.05.

**
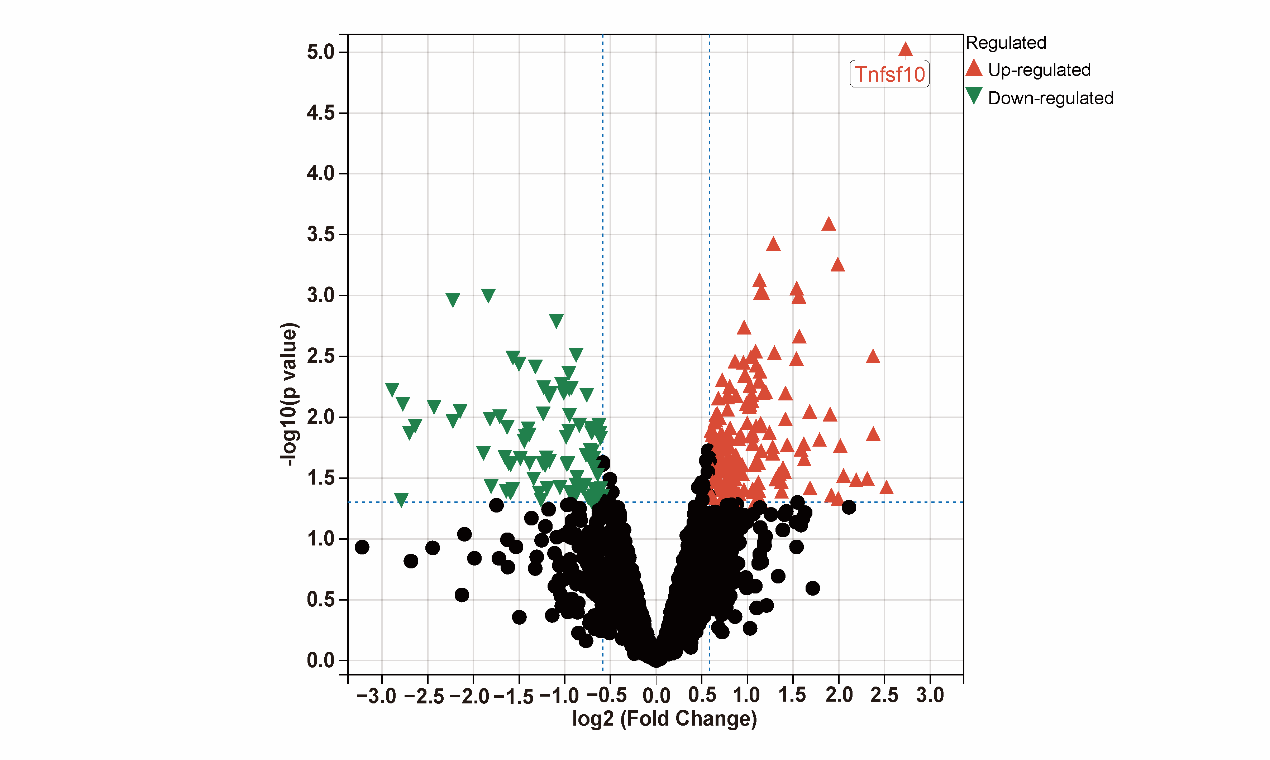
Figure S2.** Volcano plots show the differently expressed proteins of supernatants from irradiated AECII vs normal AECII.

**Figure S3.** *Trail-*cKO combined with DQ alleviated RILI. A) Representative flow **
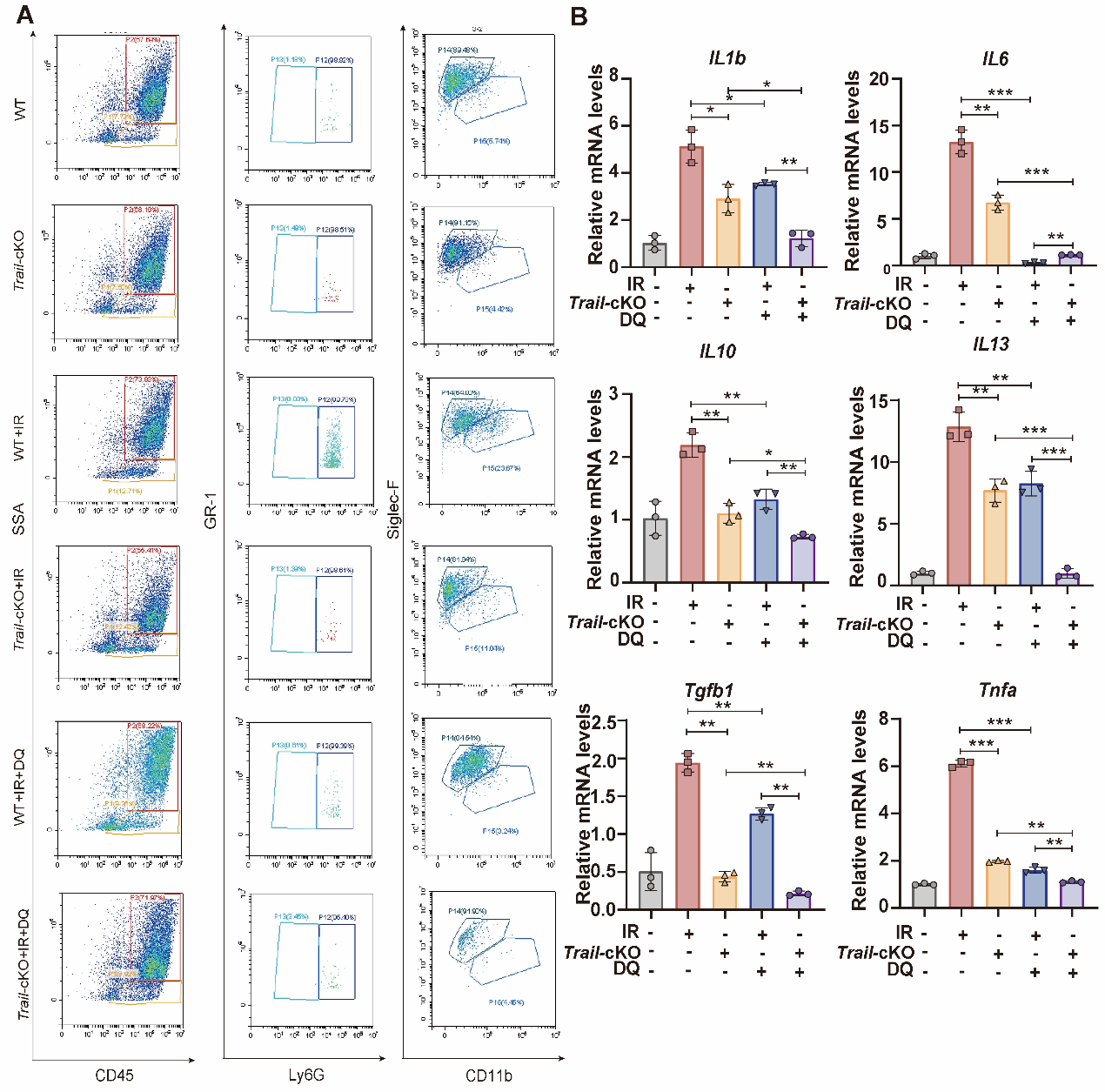
**cytometry analysis of BALF in WT, *Trail-*cKO, WT+IR, *Trail-*cKO +IR, WT +IR+DQ, *Trail-*cKO +IR+DQ groups. B) mRNA expression of cell senescence cytokines. Data shown as mean ± SEM, N = 3; *, *P* < 0.05; **, *P* < 0.01; ***, *P* < 0.001; NS, *P* > 0.05.

**
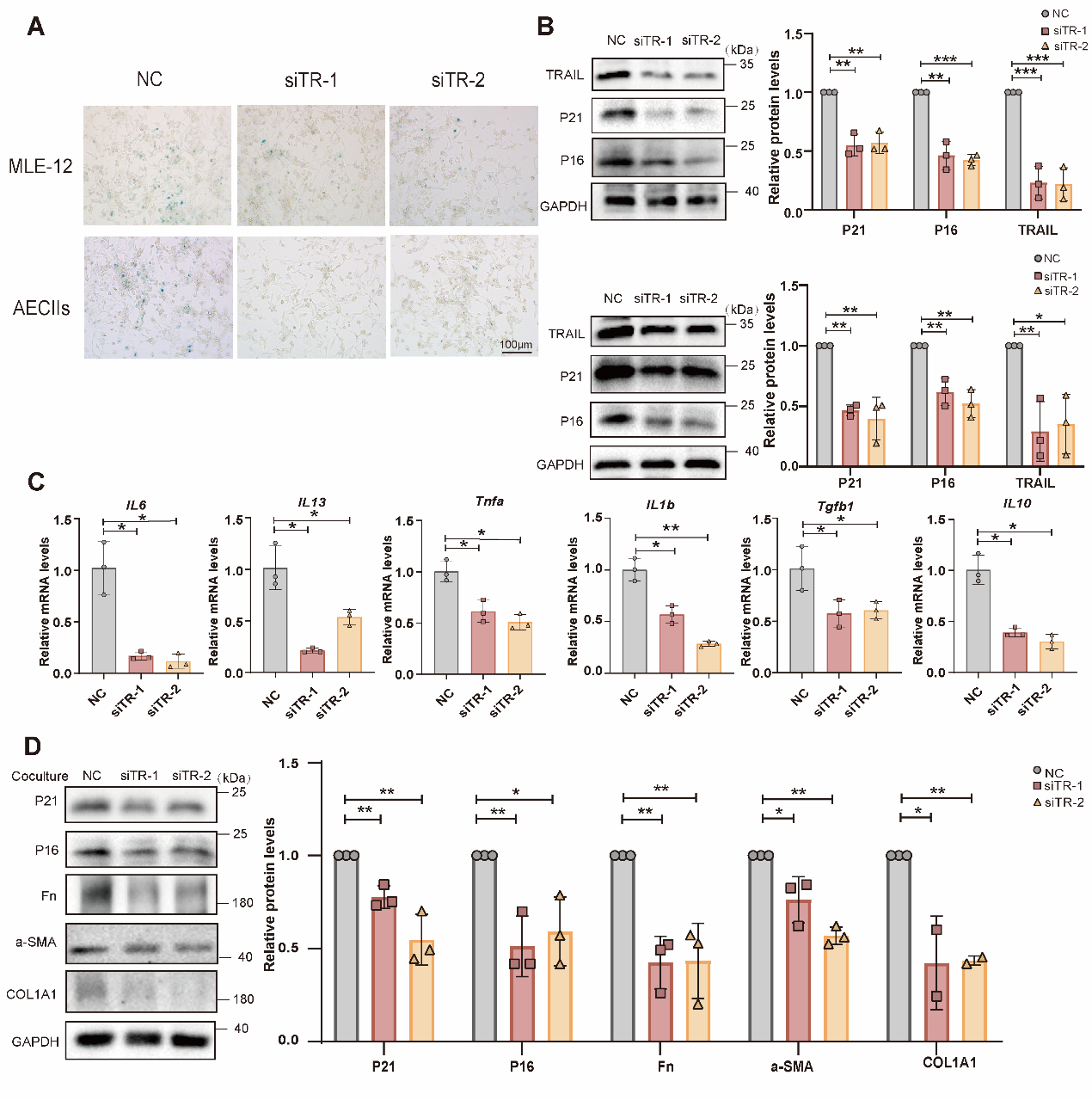
Figure S4.** Depletion of TRAIL decreased AECIIs senescence and fibrosis characteristics of fibroblast cells. A) β-Galactosidase Staining after irradiation with TRAIL knockdown. B) Protein expression of cell senescence-related molecules with TRAIL knockdown in MLE-12 cells and AECIIs. C) mRNA expression of cell senescence-related genes in MLE-12 cells. D) Protein expression of cell senescence-related molecules and fibrogenic proteins in primary lung fibroblast cells cocultured with supernatant from irradiated primary AECIIs. siTR-1, siRNA-1 for TRAIL; siTR-2, siRNA-2 for TRAIL. Data shown as mean ± SEM, N = 3; *, *P* < 0.05; **, *P* < 0.01; ***, *P* < 0.001; NS, *P* > 0.05.

**
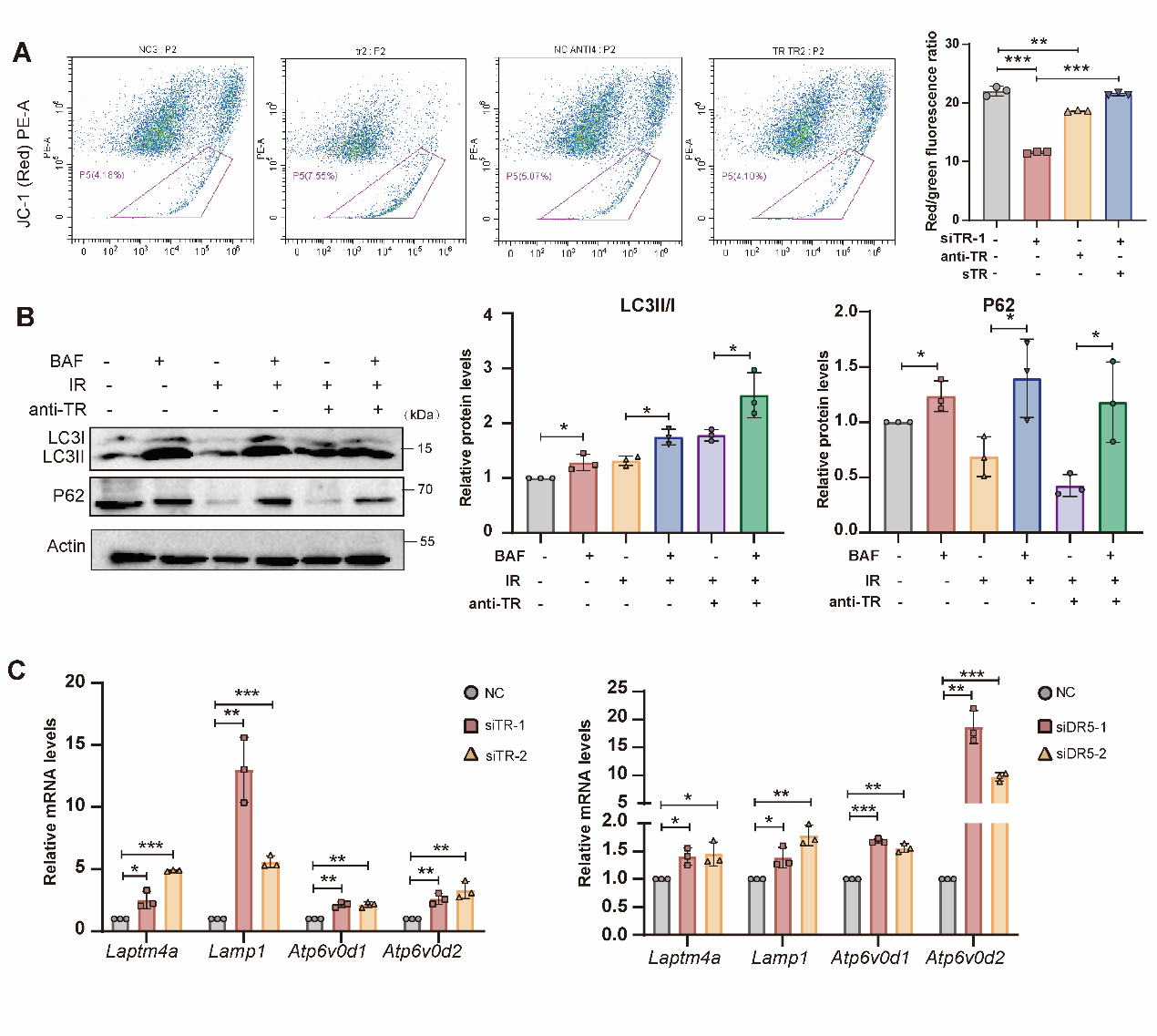
Figure S5.** TRAIL promoted cell senescence via inhibiting mitophagy in irradiated MLE-12 cells. A) JC-1 staining assays (10 Gy) under TRAIL knockdown or TRAIL antibody treatment. B) Autophagy-related protein expression of MLE-12 cells treated with BAF (400nM) for last 4 hours. C) Representative mRNA expression of lysosome function-related genes. siTR-1, siRNA-1 for TRAIL; anti-TR, TRAIL antibody. Data shown as mean ± SEM, N = 3; *, *P* < 0.05; **, *P* < 0.01; ***, *P* < 0.001.


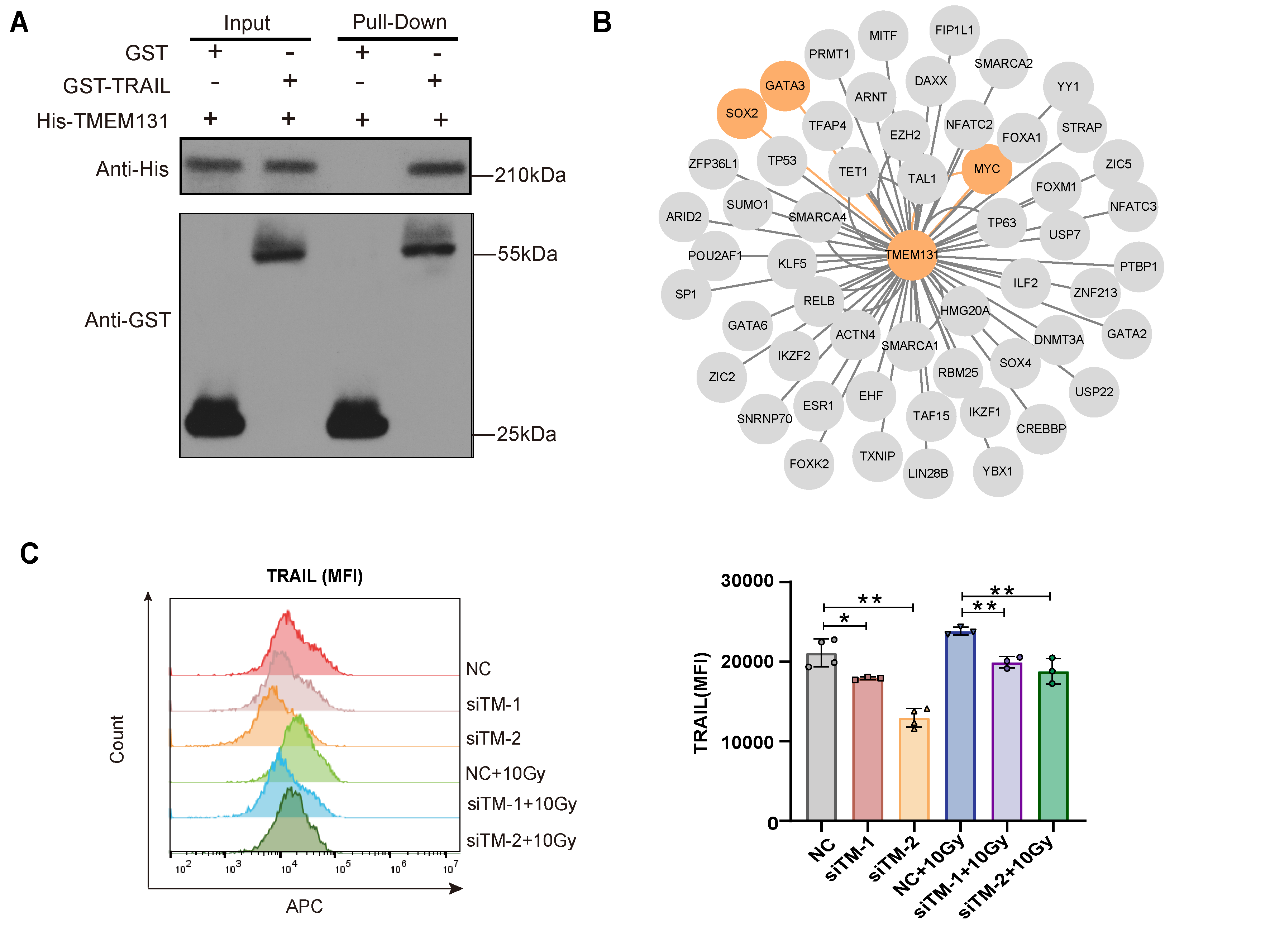
 **Figure S6.** TMEM131 regulates the expression of TRAIL in RILI. A) GST-TRAIL was capable of pulling down TMEM131 IN 293T cells. B) Upstream transcription factors of TMEM131. C) Flow cytometry analysis showing the presence of TRAIL on cell membrane with irradiation or not in TMEM131 depletion MLE-12 cells. siTM-1, siRNA-1 for TMEM131; siTM-2, siRNA-2 for TMEM131. Data shown as mean ± SEM, N = 3; *, *P* < 0.05; **, *P* < 0.01.


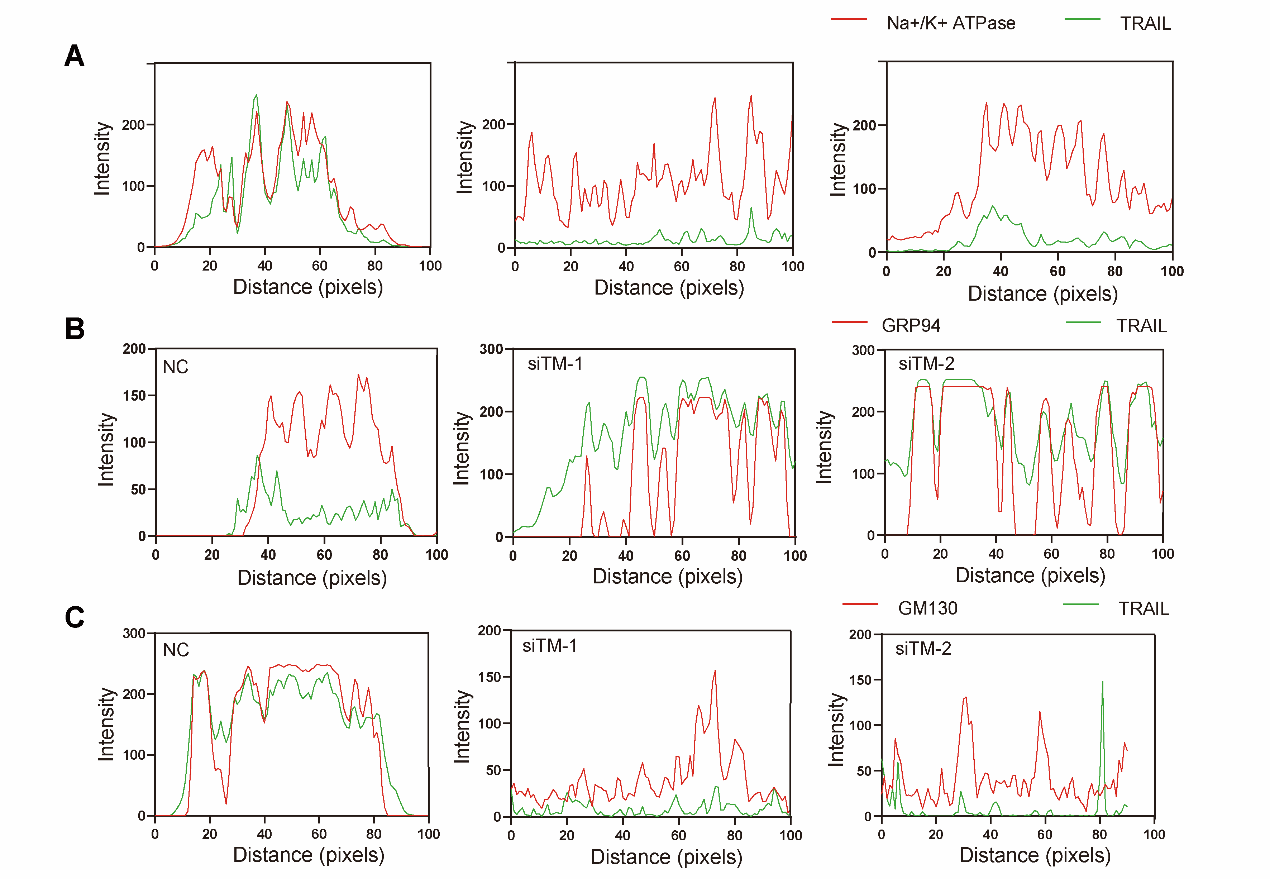
**Figure S7.** The intensity profiles of TRAIL, Na+/K+ ATP (A), GRP94 (B) and GM130 (C) in respective white dashed boxes.

**
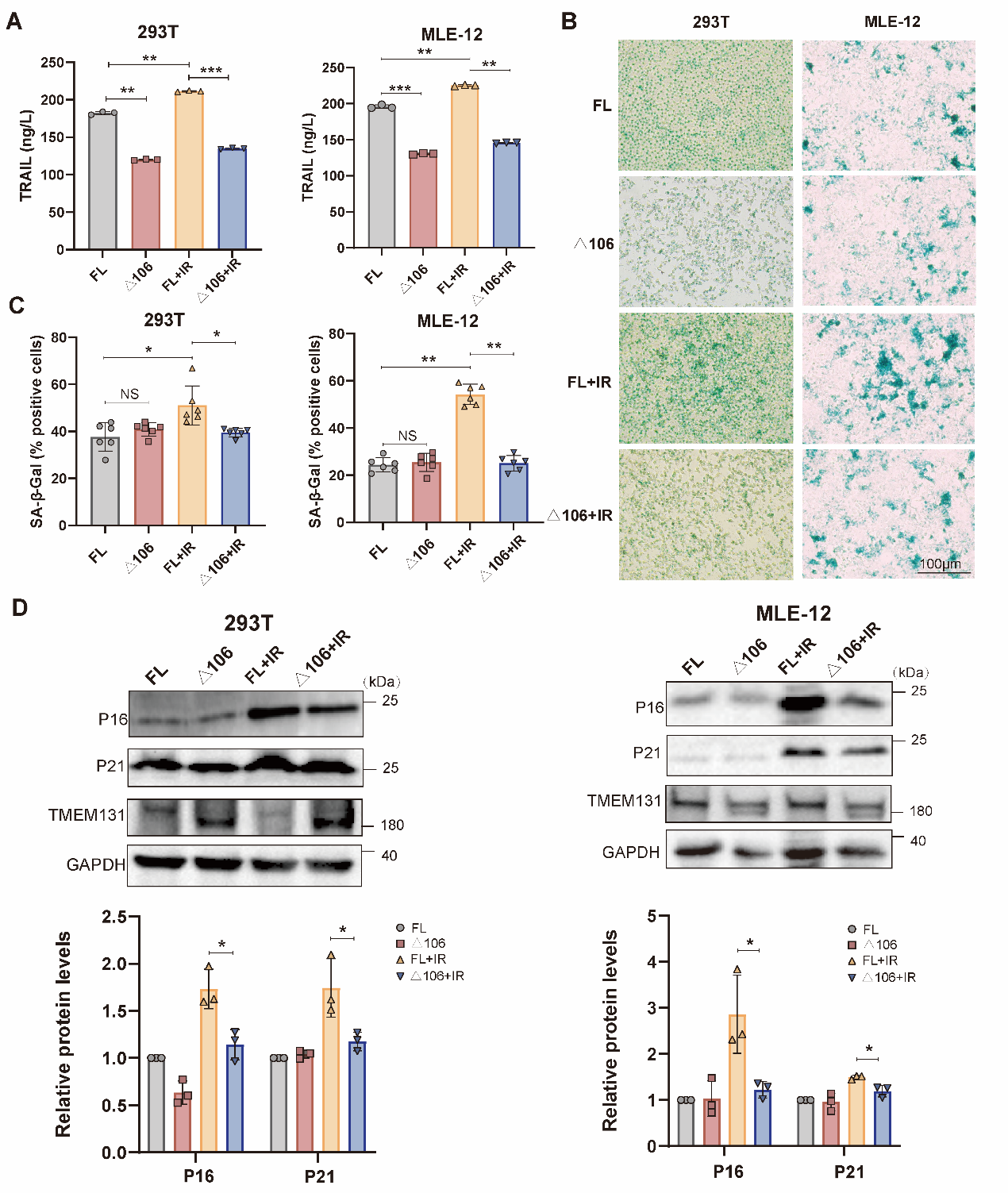
**

**Figure S8.** Disruption of TRAIL-TMEM131 interacting domain decreased TRAIL secretion and cell senescence in MLE-12 cells. A) ELISA assays showing the expression of TRAIL in supernatant from overexpressing TMEM131 or Δ106 group. B, C) β-galactosidase staining with TMEM131 or Δ106 overexpression (B) and quantitative analysis (C). D) Protein expression of cell senescence-related molecules with TMEM131 or Δ106 overexpression. Data shown as mean ± SEM, N = 3-6; *, *P* < 0.05; **, *P* < 0.01; ***, *P* < 0.001; NS, *P* > 0.05.

**Figure S9.
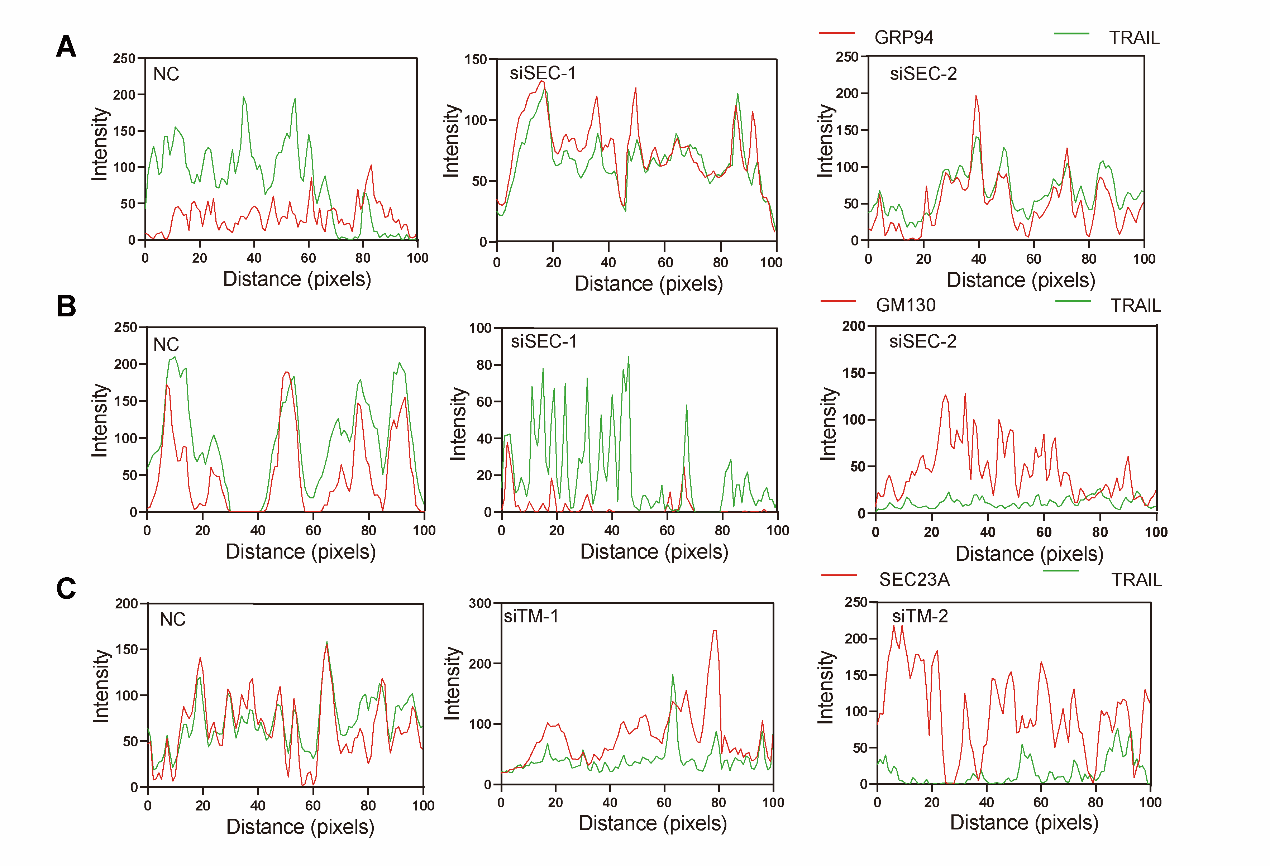
** The intensity profiles of TRAIL, GRP94 (A), GM130 (B) and SEC23A (C) in respective white dashed boxes.


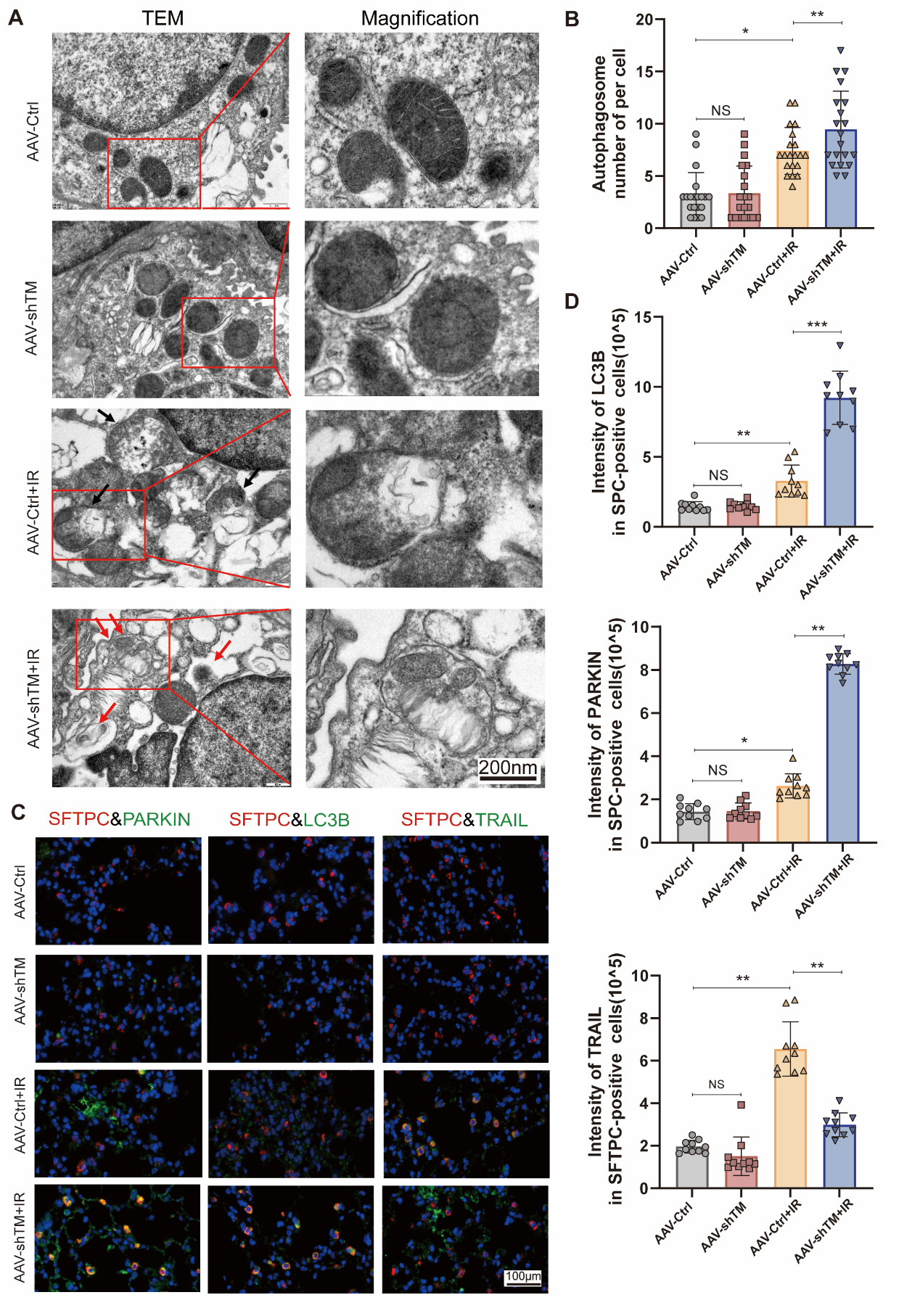


**Figure S10.** Targeting TMEM131 in AECII enhanced mitophagy influx *in vivo*. A) Representative electron microscopy images for mitophagosome in AECIIs. B) Quantitative analysis of mitophagosomes. C) Immunofluorescent co-staining of SFTPC and PARKIN (left), co-staining of SFTPC and LC3B (middle), co-staining of SFTPC and TRAIL (right). D) Quantitative analysis of co-staining intensity. Data shown as mean ± SEM, N = 10-20; *, *P* < 0.05; **, *P* < 0.01; ***, *P* < 0.001; NS, *P* > 0.05.

**Table S1.** **Clinical information of RILI patients**

| ID | Gender | Age (Year) | Tumor | Stage | Tumor location | Dose(Gy) |
| --- | --- | --- | --- | --- | --- | --- |
| 1 | Male | 56 | SCLC | ED | Right lung | 50 |
| 2 | Male | 55 | LUAD | cT1N0M0 | Right lung | 54 |
| 3 | Male | 66 | LUAD | cT3N3M1b | Right lung | 50 |
| 4 | Female | 61 | LUAD | T3N1M1a | Right lung | 45 |
| 5 | Female | 73 | SCLC | cT2N3M0 | Right lung | 50 |
| 6 | Male | 61 | LUAD | cT2bN1M0 | Left lung | 66 |
| 7 | Male | 70 | SCLC | cT2NxM1 | Left lung | 45 |
| 8 | Male | 67 | SCLC | cT4N3M1 | Left lung | 30 |
| 9 | Male | 64 | LUAD | T3N2M1 | lung | 90 |

SCLC, small cell lung cancer; LUAD, lung adenocarcinoma.

**Table S2.** **siRNA sequences**

| siRNA | sense（5'-3'） | antisense（5'-3'） |
| --- | --- | --- |
| siTRAIL-1 | GAGGAUUGUUCGAGCUAAATT | UUUAGCUCGAACAAUCCUCTT |
| siTRAIL-2 | GUGCAGUACAUCUACAAGUTT | ACUUGUAGAUGUACUGCACTT |
| siTMEM131-1 | CCAAAUCCGUAUCGAUUAATT | UUAAUCGAUACGGAUUUGGTT |
| siTMEM131-2 | GGAUGUACCAAUAACAAGUTT | ACUUGUUAUUGGUACAUCCTT |
| siSEC23A-1 | GGGCAUGUCAUUGAUAUCUTT | AGAUAUCAAUGACAUGCCCTT |
| siSEC23A-2 | GGGUGACUCUUUCAAUACUTT | AGUAUUGAAAGAGUCACCCTT |
| simDR5-1 | GUUACUGGAACAAAGACAGCCTT | GGCUGUCUUUGUUCCAGUAACTT |
| simDR5-2 | CUUUAUCUUCCUUACAGACUGTT | CAGUCUGUAAGGAAGAUAAAGTT |
| simDR5-3 | AAUGAAUAUCGCUAGAAUCUGTT | CAGAUUCUAGCGAUAUUCAUUTT |
| simDcTRAILR2-1 | UUUAGACCAGUAUUCACCAGCTT | GCUGGUGAAUACUGGUCUAAATT |
| simDcTRAILR2-2 | AAUUAUCUUUCUCUGUGAAUGTT | CAUUCACAGAGAAAGAUAAUUTT |
| simDcTRAILR2-3 | CAUAGUAGUAAAGACCUGUUCTT | GAACAGGUCUUUACUACUAUGTT |
| simDcTRAILR1-1 | AUUAGACUGGUAUUCACCAUCTT | GAUGGUGAAUACCAGUCUAAUTT |
| simDcTRAILR1-2 | UAUUCUGGUCUUUAUCACAGGTT | CCUGUGAUAAAGACCAGAAUATT |
| simDcTRAILR1-3 | CCUUGACAAAUGUACCUGAGGTT | CCUCAGGUACAUUUGUCAAGGTT |
| siEDEM2-1 | GGGACCUUCAUUGUGGAAUTT | AUUCCACAAUGAAGGUCCCTT |
| siEDEM2-2 | CCAUCCUUCUUCAGGAUAATT | UUAUCCUGAAGAAGGAUGGTT |
| siEDEM2-3 | GGCUGCCAGAAUUCUACAATT | UUGUAGAAUUCUGGCAGCCTT |
| siHRD1-1 | GAGACUUGUUUGGCCUUCATT | UGAAGGCCAAACAAGUCUCTT |
| siHRD1-2 | GGGACAACAAGGCUGUAUATT | UAUACAGCCUUGUUGUCCCTT |
| siHRD1-3 | CUGUGACAGAUGCCAUCAUTT | AUGAUGGCAUCUGUCACAGTT |

**Table S3.** **Information of antibodies**

| Antibody | Dilution | Application | Company |
| --- | --- | --- | --- |
| Purified anti-mouse CD45 (103102) | - | AECIIs | Biolegend |
| Purified anti-mouse CD32 (156402) | - | AECIIs | Biolegend |
| TRAIL (bs-1214R) | 1:1000 | WB | Bioss |
| TRAIL (bs-1214R) | 1:100 | IF | Bioss |
| GAPDH (10494-1-AP) | 1:10000 | WB | Proteintech |
| SFTPC (10774-1-AP) | 1:100 | IF | Proteintech |
| P21 (28248-1-AP) | 1:1000 | WB | Proteintech |
| P16 (10883-1-AP) | 1:1000 | WB | Proteintech |
| α-SMA (19245) | 1:1000 | WB | Cell Signaling Technology |
| Col1 (72026S) | 1:1000 | WB | Cell Signaling Technology |
| Fib (15613-1-AP) | 1:1000 | WB | Proteintech |
| DR5 (A19043) | 1:1000 | WB | Abclonal |
| mDcTRAILR2 (MAB1816-SP) | 1:1000 | WB | R&D Systems |
| mDcTRAILR1 (AF2378-SP) | 1:1000 | WB | R&D Systems |
| Actin (GB15003-100) | 1:1000 | WB | Servicebio |
| LC3B (2775S) | 1:1000 | WB | Cell Signaling Technology |
| Beclin1 (D40C5) | 1:1000 | WB | Cell Signaling Technology |
| p62 (A11483) | 1:1000 | WB | Abclonal |
| PARK2 (14060-1-AP) | 1:1000 | WB | Proteintech |
| p-mTOR (AP0978) | 1:1000 | WB | Abclonal |
| mTOR (66888-1-Ig) | 1:1000 | WB | Proteintech |
| TMEM131 (A302-129A-T) | 1:1000 | WB | Bethyl |
| TMEM131 (A302-129A-T) | 1:100 | IF | Bethyl |
| Na+/K+ ATP (14418-1-AP) | 1:100 | IF | Proteintech |
| GM130 (11308-1-AP) | 1:1000 | WB | Proteintech |
| GM130 (11308-1-AP) | 1:100 | IF | Proteintech |
| GRP94 (14700-1-AP) | 1:1000 | WB | Proteintech |
| GRP94 (14700-1-AP) | 1:100 | IF | Proteintech |
| Sec23A (A8613) | 1:1000 | WB | Abclonal |
| Sec23A (A8613) | 1:100 | IF | Abclonal |
| HA (AE008) | 1:1000 | WB | Abclonal |
| Flag (AE092) | 1:1000 | WB | Abclonal |
| Ubiquitin (10201-2-AP) | 1:1000 | WB | Proteintech |
| EDEM2 (A17181) | 1:1000 | WB | Abclonal |
| HRD1 (13473-1-AP) | 1:1000 | WB | Proteintech |

**Table S4.** **Primer sequences**

| Gene | Sequence (5'to3') | Organism |
| --- | --- | --- |
| IL1B Fp | TGCCACCTTTTGACAGTGATG | Mouse |
| IL1B Rp | TTCTTGTGACCCTGAGCGAC | Mouse |
| IL6 Fp | TAGTCCTTCCTACCCCAATTTCC | Mouse |
| IL6 Rp | TTGGTCCTTAGCCACTCCTTC | Mouse |
| IL10 Fp | AAGACCCAGACATCAAGGCG | Mouse |
| IL10 Rp | AGGCATTCTTCACCTGCTCC | Mouse |
| IL13 Fp | CAGCTCCCTGGTTCTCTCAC | Mouse |
| IL13 Rp | CCACACTCCATACCATGCTG | Mouse |
| TGFb1 Fp | ACATTGACTTCCGCAAGGAC | Mouse |
| TGFb1 Rp | CCGGGTTATGCTGGTTGTA | Mouse |
| TNFa Fp | AAGCCTGTAGCC CACGTCGTA | Mouse |
| TNFa Rp | GGCACCACTAGTTGGTTGTCTTTG | Mouse |
| TMEM131 Fp | GCCCTCCCTAGACCCAACTG | Mouse |
| TMEM131 Rp | GCTTCCAAGTAGGCTGTTCCA | Mouse |
| TRAIL Fp | TCACCAACGAGATGAAGCAGC | Mouse |
| TRAIL Rp | CTCACCTTGTCCTTTGAGACC | Mouse |
| lamp1 Fp | CCAGAGCGTTCAACATCAGC | Mouse |
| lamp1 Rp | ACAGGCTAGAGCTGGCATTC | Mouse |
| Laptm4a Fp | TGCGTTCTTTTTGCCGTCTC | Mouse |
| Laptm4a Rp | GAATCAGCCAGCCCACTTGA | Mouse |
| Atp6v0d1 Fp | CGCCACATGAGAAACCATGC | Mouse |
| Atp6v0d1 Rp | CTCAAAGCTGCCTAGCGGAT | Mouse |
| Atp6v0d2 Fp | CTGGTTCGAGGATGCAAAGC | Mouse |
| Atp6v0d2 Rp | TCCAAGGTCTCACACTGCAC | Mouse |
| actin Fp | GTGACGTTGACATCCGTAAAGA | Mouse |
| actin Rp | GCCGGACTCATCGTACTCC | Mouse |
